# Soluble CD30, the Immune Response, and Acute Rejection in Human Kidney Transplantation: A Systematic Review and Meta-Analysis

**DOI:** 10.3389/fimmu.2020.00295

**Published:** 2020-02-28

**Authors:** Mohammad Mirzakhani, Mehdi Shahbazi, Roghayeh Akbari, Ivana Dedinská, Eghlim Nemati, Mousa Mohammadnia-Afrouzi

**Affiliations:** ^1^Student Research Committee, School of Medicine, Babol University of Medical Sciences, Babol, Iran; ^2^Immunoregulation Research Center, Health Research Institute, Babol University of Medical Sciences, Babol, Iran; ^3^Cellular and Molecular Biology Research Center, Health Research Institute, Babol University of Medical Sciences, Babol, Iran; ^4^Kidney Transplantation Center, Shahid Beheshti Hospital, Babol University of Medical Sciences, Babol, Iran; ^5^Surgery Clinic and Transplant Center, University Hospital Martin and Jessenius Faculty of Medicine Comenius University, Martin, Slovakia; ^6^Nephrology and Urology Research Center, Baqiyatollah University of Medical Sciences, Tehran, Iran

**Keywords:** kidney transplantation, acute rejection, biomarker, Soluble CD30, immune system

## Abstract

Soluble CD30 (sCD30) is considered to be a marker for the activated immune system in which T cells can damage the allograft. Some studies reported that post-transplant sCD30 can predict early acute rejection and can thereby be used as a biomarker to detect acute rejection. However, several other studies found no relation between post-transplant sCD30 and acute rejection. This meta-analysis study aims to answer this main question of whether sCD30 can help clinicians to monitor transplant recipients. The electronic databases, including PubMed, Web of Science, ProQuest, Embase, Scopus, Google Scholar, the gray literature, and the key journals, were searched for observational studies from 1 January 1990 up to 30 April 2018. Eighteen studies, with a total of 1,453 patients, were included in this paper. With regard to the different measurement times, post-transplant sCD30 was separately analyzed and divided into five groups (i.e., 1, 2, 3, 4 week, and 1 month post-transplant sCD30). All groups indicated a strong association between sCD30 and the acute rejection. The standardized mean difference (SMD) is 1.22 in 1 week, 0.77 in 2 week, 1.11 in 3 week, 1.27 in 4 week, and 0.71 in 1 month groups. The association between sCD30 and acute rejection was consistent across all the subgroup analyses. We found that post-transplant sCD30 had a strong association with acute kidney rejection. We also found that the deceased donors had more association with the high amount of sCD30 than living donors in patients with acute rejection. Finally, we realized that donor type was an important factor leading to the heterogeneous results in the primary studies.

## Introduction

Kidney transplantation is the best treatment for patients suffering from end-stage renal disease (ESRD), which improves the patients' life quality and reduces costs. However, acute rejection episodes (AREs), which are caused by immunological responses, reduce the allograft survival and can further contribute to chronic rejection (1).

There is not any non-invasive biomarker to detect early rejection or monitor patients' status after kidney transplantation. Early diagnosis of acute rejection may help the clinicians to manage the patients' status better (2).

Serum creatinine and proteinuria, which are considered to be conventional approaches to reflect the allograft function, are a non-specific method. On the other hand, the biopsy, the gold standard procedure for confirming the rejection, has several limitations. For instance, it is an invasive procedure with subsequent complications, such as infection, and thereby cannot be continuously used to monitor patient status (2).

The role of soluble CD30 (sCD30) was evaluated in some studies in order to determine whether sCD30 can predict early acute rejection or can be considered as a biomarker to monitor early patients' status after transplantation.

The CD30 molecule is a transmembrane glycoprotein that belongs to the tumor necrosis factor and nerve growth factor receptor superfamily, and it has a molecular weight of 120-kDa. It is expressed on natural killer cells (3), dendritic cells, regulatory T cells, as well as CD4^+^ and CD8^+^ activated T cells but not on resting T cells (4, 5).

Activated effector and memory T cells express CD30 upon polyclonal activation or alloimmune stimulation. The metalloproteinases ADAM17 and ADAM10 cleave the membrane-bound CD30 extracellular domain, and sCD30 is then released by activated CD30^+^ T cells (particularly memory CD4^+^ T cells) into the bloodstream. Thus, they can be easily measured by ELISA (3, 6–8).

Although the function of CD30 remains unclear, participation in signal transduction, which leads to fast NF-κB activation, and regulation of the balance between TH1/TH2 responses have been suggested for the CD30 function (3, 5). It was reported that regulatory T cells (Tregs) could suppress memory CD8^+^ T cell through a CD30/CD30 ligand (CD153) interaction, which could contribute to allograft survival (9). However, sCD30 has more affinity for the CD30 ligand and prevents such regulatory function of Tregs. A high concentration of sCD30 may consequently imply the presence of effector or memory T cells (activated immune system) and a high alloreactivity condition (10).

Several studies have shown that high post-transplant serum levels of sCD30 are associated with AREs and poorer graft survival (8, 11, 12). Moreover, it was reported that sCD30 is related to antibody-mediated rejection (ABMR). During allogeneic stimulation of T cells, CD30 is upregulated on the memory CD4^+^ and CD8^+^ T cells. The CD30 will thus release into the blood, and the increased level of sCD30 may consequently imply the activation of T cells and subsequent allograft damage (10, 13, 14). By contrast, some studies indicated that there is no correlation between the sCD30 serum concentration and the occurrence of acute rejection or late acute rejection (15–17). Moreover, CD30^+^ T cells are available at inflammatory sites of several autoimmune diseases, such as atopic dermatitis, rheumatoid arthritis, and systemic sclerosis (18).

We cannot definitely suggest that the sCD30 can predict rejection episodes or graft loss, but, on the other hand, we should not ignore its role as a predictor of AREs and its association with poorer graft survival, which was established by Süsal et al. (10, 13, 14). Therefore, we conducted a systematic review and meta-analysis to assess whether sCD30 is related to acute rejection in kidney transplant recipients.

## Method

This systematic review and meta-analysis was prospectively registered with the National Institute for Health Research PROSPERO system (registration No. CRD42018101993). It is reported via the Reporting Checklist for Meta-analyses of Observational Studies (MOOSE) (19) and Preferred Reporting Items for Systematic Reviews and Meta-analyses (PRISMA) statement (20) (Supplementary Table 1).

### Eligibility Criteria

#### Type of Study

We selected all types of observational studies in this paper, including cross-sectional, case-control, or cohort studies, with all subtypes in each study. Case-report, case-series, interventional studies (randomized and non-randomized), narrative reviews, animal studies, and letters were excluded from this study. In addition, we excluded studies that did not separate acute and chronic rejection patients.

#### Type of Participant

Patients with normal allograft function and patients with acute biopsy-proven rejection (BPR), whose post-transplant sCD30 levels were assessed, were enrolled in this systematic review and meta-analysis if they fulfilled several criteria: (i) patients with receiving either a living donor or deceased donor kidney transplant, (ii) patients with receiving either one or more than one kidney graft, (iii) patients who had ≥6 months of follow-up, and any age (or one of the subtypes of age), any gender (or at least one of them), and any demographic data were included in this review. The rejection was confirmed by a biopsy. However, the grade—the grade of rejection—was part of the missing data; the studies did not report it or the number of studies made, and this was not enough to carry out the subgroups.

#### Outcome

The outcome was acute rejection, which was proved by a biopsy as a gold standard test. Acute rejection was considered to be a type of cell-mediated or antibody-mediated rejection of any grade.

### Search Strategy

We searched several sources without language limitation to achieve the research goal. These sources include electronic databases, such as PubMed, Web of Science, ProQuest, Embase, Scopus, Google Scholar, and gray literature (conference/congress paper and thesis), the key journals (transplantation and transplantation proceedings), and the reference lists of included primary research. The primary studies are from 1 January 1990 up to 30 April 2018. The search syntax, with distinctive electronic databases, is shown in Supplementary Tables 2–6.

For achieving great syntax, the main search terms, which were “CD30 antigen,” “kidney transplantation,” “graft rejection,” and “acute graft rejection,” were searched for in MeSH and EMtree. We also used the free-text method to achieve great syntax.

### Study Selection

We exported our search output into the End-note software and deleted duplicated studies (only one version of primary studies was kept). The screening step (included/probably included vs. excluded primary researches) was performed according to the title and abstract. Then two reviewers, via full-text assessment, independently conducted the selection/confirmation process (included vs. excluded primary researches) according to the eligibility criteria. Any discordance was resolved by consensus.

### Risk of Bias Assessment

Two reviewers independently conducted a quality assessment of primary studies by using a modified version of the quality assessment checklist for observational studies (Newcastle-Ottawa scale) (21) (Supplementary Text 1). Any disagreement was resolved by consensus.

## Data Extraction

A data extraction form was developed by expert opinion and also by using papers included in this study. Several data were extracted from each study: first author, publication year, country of origin, maintenance therapy, induction therapy (IT), donor type (living vs. deceased), the number of transplanted kidneys, the age of rejection patients, the panel reactive antibody (PRA), and the cold ischemia time (CIT) of rejection patients. The main data for meta-analysis were the number of patients and mean ± SD of sCD30 levels (n_1_m_1_s_1_ n_2_m_2_n_2_) in each group (rejection vs. stable). The main data of meta-analysis were categorized into five groups: 1 week-post-transplant sCD30 group, 2 week-post-transplant sCD30 group, 3 week-post-transplant sCD30 group, 4 week-post-transplant sCD30 group, and 1 month-post-transplant sCD30 group. In addition, there was another group within the 1 week-post-transplant sCD30 group in which sCD30 was measured before or at the time of the acute rejection.

Two reviewers independently extracted information via data extraction form, and discordance was resolved by consensus. For incomplete data, we contacted the corresponding authors of the studies.

## Data Synthesis and Analyses

First of all, we selected the concordant valid effect size (standardized mean difference [SMD]) on the basis of the data extraction form; and then, according to the methodological similarities between final eligible papers, the appropriate combination model (fixed-effects model) was selected (22) and the combined effect size was plotted via forest plot.

We assessed the heterogeneity of the studies by using a Q Cochrane test and its *p*-value and *I*^2^ index (23). The severe heterogeneity was considered *I*^2^ > 50%.

### Publication Bias Assessment

We used Funnel plots to assess publication bias visually, and we used Begg's and Egger's tests (24, 25) to assess the bias statistically. We also used the Trim and Fill method to confirm the previous assessment of publication bias (26).

### Additional Analyses

According to the above statements (see Data Extraction section), the main data for meta-analysis were divided into six groups. However, regarding the number of studies in each group, only data of 1 week-post-transplant sCD30 and 2 week-post-transplant sCD30 levels were considered for subgroup analyses.

#### Subgroup Analyses

In subgroup analyses, studies with higher similarity were analyzed together. Subgroup analyses were conducted to find out potential sources of heterogeneity. Another purpose of the subgroup analyses was to know whether the effect size was influenced by different subgroup analyses.

Subgroup analyses were performed as follows:

##### The number of transplanted kidneys

Here, patients with one kidney allograft were compared with patients who received more than one allograft.

##### Donor type

In this paper, studies used a living or deceased donor. Thus, this subgroup was divided into two categories, i.e., living and deceased.

##### Induction therapy

Patients who received IT were compared with patients who did not receive it.

##### Methodological quality of studies

The high methodological quality was considered an NOS score ≥6 vs. those with low methodological quality, where the NOS score ≤5.

##### Panel reactive antibody

A PRA >10% was compared with a PRA <10% in this study.

#### Meta-Regression Analyses

Since the data regarding the age and CIT in rejection patients were not enough for subgroup analyses, we conducted meta-regression analyses on these data to find out their association with sCD30 and acute rejection.

#### Sensitivity Analysis

The one-out-remove method was done for sensitivity analysis. In this method, the pooled estimate is recalculated after removing the effect of each study. This method was used to know whether there was any difference between the different results. If yes, how much does the recalculated pooled estimate change?

This study was analyzed by using STATA version 12 (StataCorp, College Station, TX, USA).

## Results

### Study Selection

**Figure 1:**
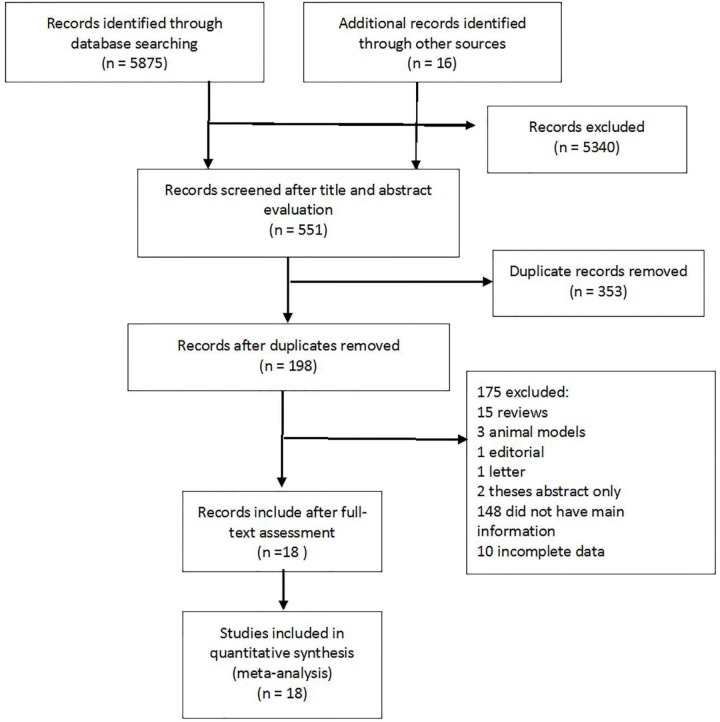
PRISMA flow chart. The flow chart summarizes study identification and selection.

A total of 5,875 records were identified via an electronic search, and 16 additional records were added from the gray literature search. Figure 1 displays the PRISMA flow chart that shows the literature search procedure. Based on the title and abstract, after screening the 5,891 records, 551 records remained. Then, after removing duplicated records, 198 records remained. Next, after reviewing the full text, 175 records were excluded, and, finally, the studies, which had the relevant data, remained for meta-analysis. Therefore, 18 studies (8, 11, 12, 15–17, 27–38) and a total of 1,453 patients were included in the final meta-analysis.

### Study Characteristics

The studies were included from 2005 to 2018 and had a cohort study design. The main data for meta-analysis were post-transplant sCD30 levels in patients with acute rejection, which were compared with patients without rejection (stable patients). The sCD30 was measured by the same ELISA kit in all but three studies, of which one of them did not report the method of sCD30 measurement.

Of 18 studies included in this paper, 13 studies had a 1 week or 2 week-post-transplant sCD30 level and acute rejection status; three studies had a 3 week or 4 week-post-transplant sCD30 level and acute rejection status; and five studies had a 1 month-post-transplant sCD30 level and acute rejection status.

In addition, there were six studies within the 1 week-post-transplant sCD30 group in which sCD30 was measured before or at the time of the acute rejection. Thus, the main data for meta-analysis were divided into six groups, and each of them was separately analyzed by the author. Ten studies provided donor type (living vs. deceased), and six studies used IT. Moreover, 9, 15, 9, and 6 studies had the data of transplanted kidney number, PRA, age, and CIT, respectively (Table 1). All the studies used the same maintenance therapy. The detailed characteristics of included studies are reported in Supplementary Table 7.

**Table 1:** Characteristics of the 18 included studies.

**Author**	**Transplanted kidneys No**.	**Donor type**	**PRA**	**Quality score**	**Study type**	**Rejection patient No. 1 week**	**Rejection patient mean. 1 week**	**Rejection patient SD. 1 week**	**Stable patient No. 1 week**	**Stable patient mean. 1 week**	**Stable patient SD. 1 week**
Ayed et al. (8)	One transplant recipients	Both	–	6	Cohort	18	188.15	44.56	34	171.6	81.69
Wang et al. (27)	–	Deceased	<10%	7	Cohort	39	88.4	20.6	202	43.2	19.1
Hamer et al. (15)	Several transplant recipients	–	–	6	Cohort	–	–	–	–	–	–
Wang et al. (28)	One transplant recipients	Deceased	<10%	7	Cohort	11	95	43	59	25	20
Dong et al. (29)	Several transplant recipients	Deceased	>10%	6	Cohort	49	92	27	171	41	20
Slavcev et al. (30)	One transplant recipients	Deceased	>10%	6	Cohort	–	–	–	–	–	–
Solgi et al. (31)	–	Living	<10%	6	Cohort	9	63.2	41.1	31	41.6	33.79
de Holanda et al. (32)	–	Living	>10%	6	Cohort	–	–	–	–	–	–
Halim et al. (33)	–	Both	>10%	6	Cohort	9	29.1	12	28	47.8	55
Solgi et al. (34)	–	–	<10%	5	Cohort	6	59.5	38.9	14	30.9	14.9
Yang et al. (11)	–	–	<10%	4	Cohort	20	107	68.1	38	23	20.58
Abbas et al. (16)	One transplant recipients	Living	<10%	6	Cohort	8	28.75	15.63	42	20.92	11.7
Trailin et al. (35)	Several transplant recipients	Both	–	7	Cohort	11	23.07	5.38	23	26.42	8
Domingues et al. (12)	Several transplant recipients	Living	>10%	7	Cohort	11	60.75	24	52	40	53
Kamali et al. (36)	–	Living	>10%	7	Cohort	–	–	–	–	–	–
Sengul et al. (37)	–	Both	<10%	7	Cohort	–	–	–	–	–	–
Nafar et al. (38)	One transplant recipients	Living	>10%	8	Cohort	23	112.3	99.47	152	80.95	74.94
Azarpira et al. (17)	–	Both	<10%	6	Chort	18	81.2	41.46	30	78.64	31.16

*No, Number; PRA, Panel reactive antibody; SD, Standard deviation*.

The result of the quality analysis of the 18 studies is reported in Supplementary Table 8. The NOS maximum score for each study is nine. Studies with an NOS score ≥6 were considered high, and those with ≤5 were considered of low methodological quality (39).

### The Relation Between Post-transplant sCD30 Levels and Acute Kidney Rejection

Regarding the various measurement times of sCD30 after transplantation, we decided to create a condition in which studies with similar measurement times were analyze together.

#### The 1 Week-Post-transplant sCD30 Level and Acute Rejection Status

**Figure 2:**
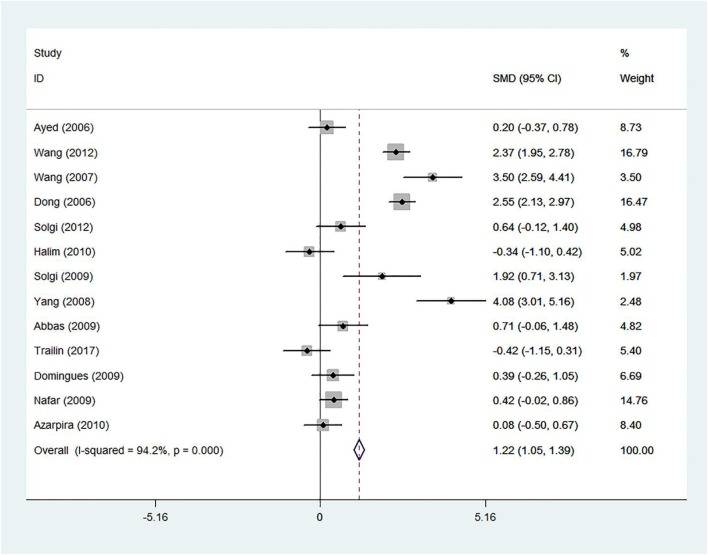
The 1-week-post-transplant sCD30 and acute rejection. The association between sCD30 and acute rejection during the first week after transplantation. SMD, Standardized mean difference; CI, Confidence interval.

The forest plot showed a strong association between the sCD30 levels, which were measured during the first week after transplantation and acute rejection (SMD 1.22; 95% confidence interval [CI] 1.05–1.39; *I*^2^ = 94.2%) (Figure 2).

##### Publication bias assessment

**Figure 3:**
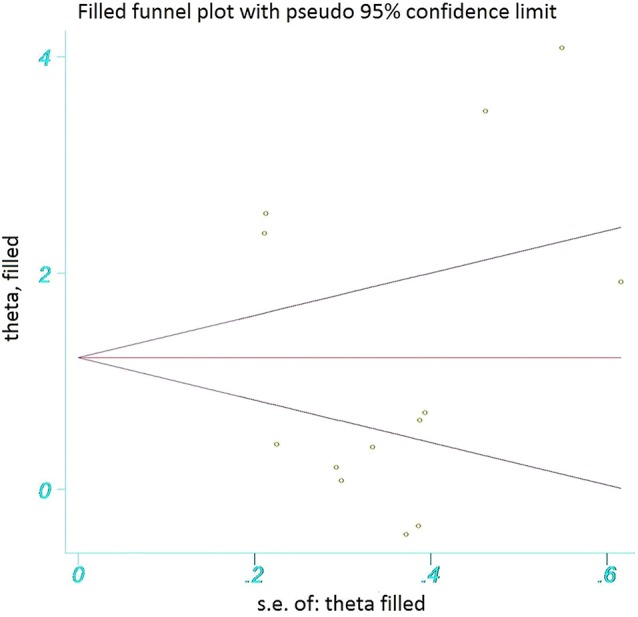
The funnel plot assessing publication bias. The funnel plot shows that no extra studies were added to this study.

The funnel plot showed a homogeneous pattern as well as the absence of publication bias (Supplementary Figure 1). The Begg's and Egger's test demonstrated non-considerable publication bias (*p* = 0.583 and *p* = 0.758), respectively. In addition, the Trim and Fill method showed no added study and confirmed the results of two previous methods (Figure 3).

##### Subgroup analyses

Because of severe heterogeneity, further analyses were performed based on subgroup analyses to find out potential sources of heterogeneity. Another purpose of the subgroup analyses was to understand whether the total results were consistent with the subgroup results. Regarding the data of primary studies, they were only performed following the subgroup analyses, and their results are summarized in Table 2.

**Table 2:** Subgroup analyses on the 1 week-post-transplant sCD30 and acute rejection.

**Subgroup variable**	**Category**	**Number of studies**	**Pooled estimate**	**95% CI**	***I*^**2**^**
Number of transplanted kidneys	One transplant recipients	4	1.35	1.02–1.69	87%
	Several transplant recipients	2	0.18	−0.11–0.47	0.0%
Donor type	Deceased	4	0.81	0.61–1.01	88%
	Living	5	0.50	0.19–0.80	75%
Methodological quality	High NOS ≥6	12	0.74	0.58–0.90	87%
	Low NOS <6	1	3.49	1.84–5.13	–
PRA	<10%	6	1.14	0.88–1.40	55%
	>10%	6	0.41	0.21–0.62	85%

*CI, Confidence interval; I^2^, I-squared; NOS, Newcastle-Ottawa Scale; PRA, Panel reactive antibody*.

##### The number of transplanted kidneys

Of 13 studies in the 1 week-post-transplant meta-analysis, only seven studies provided the number of transplanted kidneys for their study subjects. The results in the one-transplant-recipient subgroup (SMD 0.74; 95% CI 0.44–1.04; *I*^2^ = 92.7%) and several-transplant-recipient subgroup (SMD 1.48; 95% CI 1.17–1.80; *I*^2^ = 96.8%) were consistent with the results of the 1 week-post-transplant sCD30 level. However, this analysis did not justify the heterogeneity (Supplementary Figure 2).

##### Donor type

**Figure 4:**
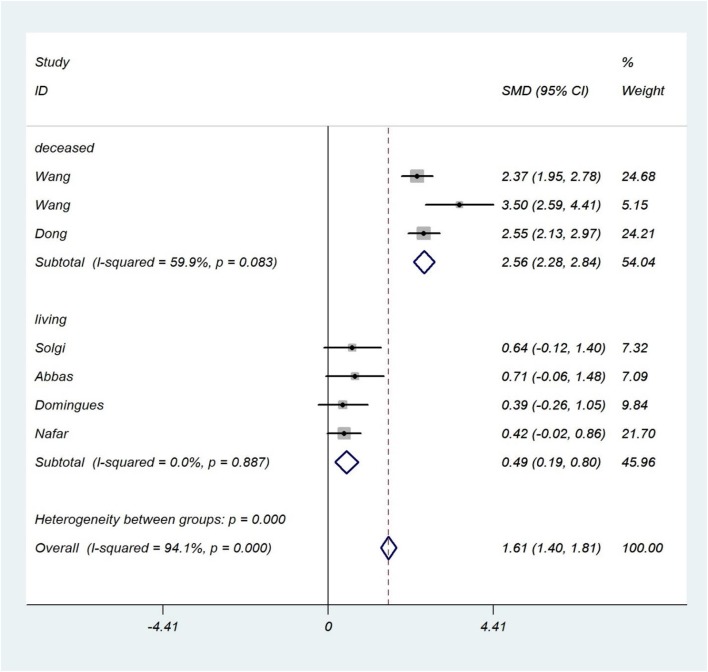
Donor type subgroup analysis. The forest plot shows the association between sCD30 and acute rejection during the first week after transplantation regarding the donor type subgroup. SMD, Standardized mean difference; CI, Confidence interval.

Of 13 studies in the 1 week post-transplant meta-analysis, 11 studies reported the type of donor. In this subgroup analysis, we had two categories (see section Additional Analyses and Donor Type). The results in the living subgroup (SMD 0.49; 95% CI 0.19–0.80; *I*^2^ = 0.0%) and deceased subgroup (SMD 2.56; 95% CI 2.28–2.84; *I*^2^ = 60%) were consistent with the results of the 1 week-post-transplant sCD30 level (Figure 4).

##### Induction therapy

**Figure 5:**
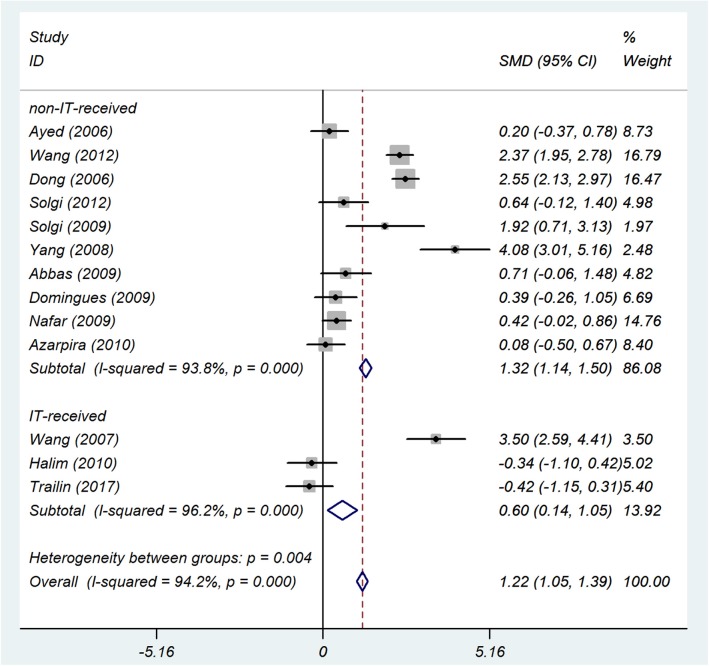
IT subgroup analysis. The forest plot shows the association between sCD30 and acute rejection during the first week after transplantation regarding the IT subgroup. SMD, Standardized mean difference; CI, Confidence interval.

Among 13 studies in the 1 week-post-transplant meta-analysis, three studies used IT. In this subgroup analysis, we had two categories (see section Additional Analyses and IT). For studies that used IT, the results were SMD 0.6; 95% CI 0.14–1.05; *I*^2^ = 96%, and for studies that did not use IT, the results were SMD 1.32; 95% CI 1.14–1.50; *I*^2^ = 94% (Figure 5).

##### Methodological quality of studies

Both high and low methodological qualities demonstrated consistent results (SMD 1.13; 95% CI 0.96–1.30; *I*^2^ = 94.3% vs. SMD 3.12; 95% CI 2.32–3.93; *I*^2^ = 85.4%, respectively). However, because there were only two studies in the low methodological quality subgroup, this result is inconclusive (Supplementary Figure 3).

##### Panel reactive antibody

Of 13 studies in the 1 week-post-transplant meta-analysis, 11 studies had the PRA data of patients. Although there was severe heterogeneity, the results of both a PRA <10% and PRA >10% (SMD 1.70; 95% CI 1.45–1.96; *I*^2^ = 93.1% vs. SMD 1.14; 95% CI 0.88–1.40; *I*^2^ = 96%, respectively) were consistent with the results of the 1 week-post-transplant sCD30 level and acute rejection status (Supplementary Figure 4).

##### Meta-regression analysis

**Figure 6:**
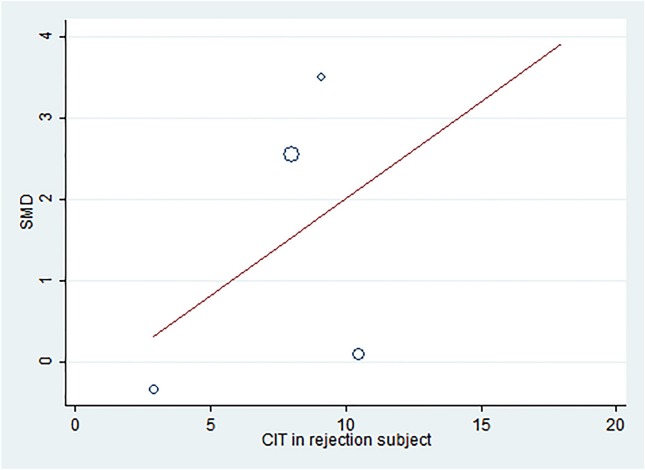
Meta-regression plot of CIT in rejection subjects. The meta-regression plot shows a positive correlation between the increased CIT and the effect of 1 week-post-transplant sCD30. SMD, Standardized mean difference; CIT, Cold ischemia time.

Meta-regression was performed on the age and CIT of patients with rejection. Age of patients with rejection did not show considerable relation to the strong effect of sCD30 (Supplementary Figure 5). However, meta-regression on CIT revealed a positive correlation between the high time of CIT and the stronger effect of sCD30 (Figure 6).

##### Sensitivity analysis

**Figure 7:**
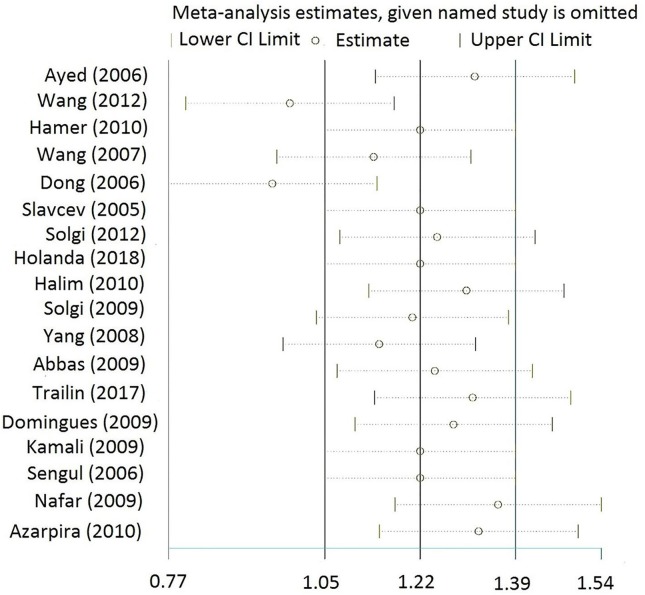
Sensitivity analysis for the 1 week-post-transplant group. Sensitivity analysis shows that two studies (27, 29) have different results. CI, Confidence interval.

The sensitivity analysis displayed that two papers (27, 29) had remarkably different results (Figure 7). However, when these studies were separately omitted, the pooled estimate did not significantly change (Supplementary Table 9).

#### The 1 Week-Post-transplant sCD30 Level and Acute Rejection Status (sCD30 Was Measured Before or at the Time of the Acute Rejection)

**Figure 8:**
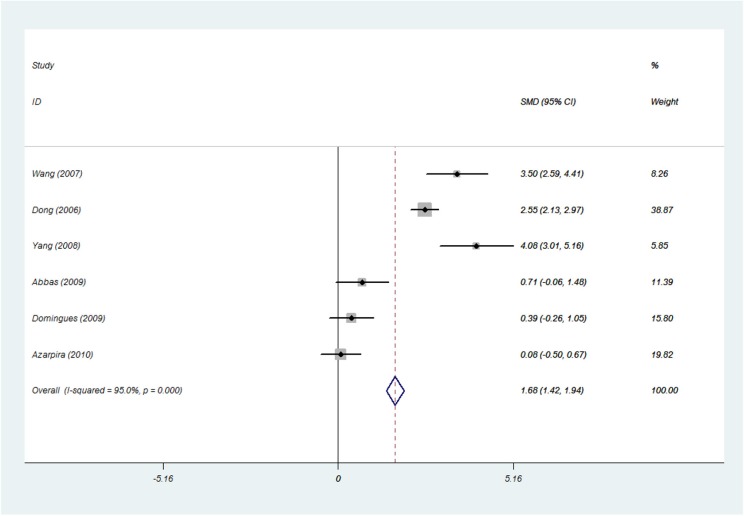
The 1 week-post-transplant sCD30 level and acute rejection status (sCD30 was measured before or at the time of acute rejection). The association between the sCD30 and the risk of acute rejection during the first weeks after transplantation. SMD, Standardized mean difference; CI, Confidence interval.

Among 13 studies in the 1 week-post-transplant sCD30 group, six studies were found to have sCD30 data, which were measured before or at the time of the acute rejection. The forest plot (SMD 1.68; 95% CI 1.42–1.94; *I*^2^ = 95%) showed a strong association between the sCD30 and risk of the acute rejection occurrence (Figure 8).

#### The 2 Week-Post-transplant sCD30 Level and Acute Rejection Status

**Figure 9:**
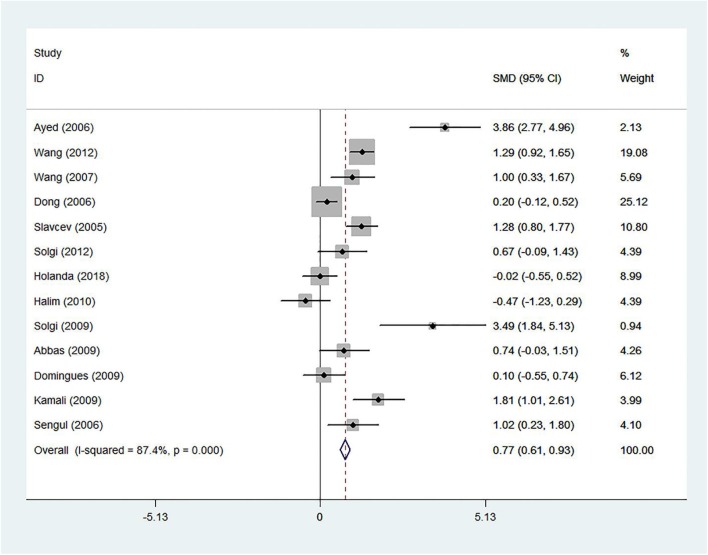
The 2 week-post-transplant sCD30 and acute rejection. The association between the sCD30 and the acute rejection during the first 2 weeks after transplantation. SMD, Standardized mean difference; CI, Confidence interval.

Thirteen studies had data of the 2 week-post-transplant sCD30 level, which meta-analysis was performed on them. The results of forest plot (SMD 0.77; 95% CI 0.61–0.93; *I*^2^ = 87.4%) indicated a strong association between the sCD30 and acute rejection (Figure 9) as well as the results of the 1 week-post-transplant sCD30. The following subgroup analyses were performed, and their results are summarized in Table 3.

**Table 3:** Subgroup analyses on the 2 week-post-transplant sCD30 and acute rejection.

**Subgroup variable**	**Category**	**Number of studies**	**Pooled estimate**	**95% CI**	***I*^**2**^**
Number of transplanted kidneys	One transplant recipients	4	0.74	0.44–1.04	93%
	Several transplant recipients	3	1.48	1.17–1.80	97%
Donor type	Deceased	3	2.56	2.28–2.84	60%
	Living	4	0.49	0.19–0.80	0.0%
Methodological quality	High NOS ≥6	11	1.13	0.96–1.30	94%
	Low NOS <6	2	3.12	2.32–3.93	85%
PRA	<10%	7	1.70	1.45–1.96	93%
	>10%	4	1.14	0.88–1.40	96%

*CI, Confidence interval; I^2^, I-squared; NOS, Newcastle-Ottawa Scale; PRA, Panel reactive antibody*.

##### The number of transplanted kidneys

In the one-transplant-recipient subgroup, results demonstrated a strong association between sCD30 and acute rejection (SMD 1.35; 95% CI 1.02–1.69; *I*^2^ = 87.4%), and these were consistent with the results of the 2 week-post-transplant sCD30 level and acute rejection. The several-transplant-recipient subgroup showed weak association with acute rejection (SMD 0.18; 95% CI −0.11–0.47; *I*^2^ = 0.0%), but, because there were only two studies in this subgroup, this result is inconclusive (Supplementary Figure 6).

##### Donor type

The results of the living subgroup indicated a reasonable association between sCD30 and acute rejection (SMD 0.50; 95% CI 0.19–0.80; *I*^2^ = 75%). The deceased subgroup showed a strong association between sCD30 and acute rejection (SMD 0.81; 95% CI 0.61–1.01; *I*^2^ = 87.8%). These results were consistent with the results of the 2 week-post-transplant sCD30 level and acute rejection (Supplementary Figure 7).

##### Induction therapy

**Figure 10:**
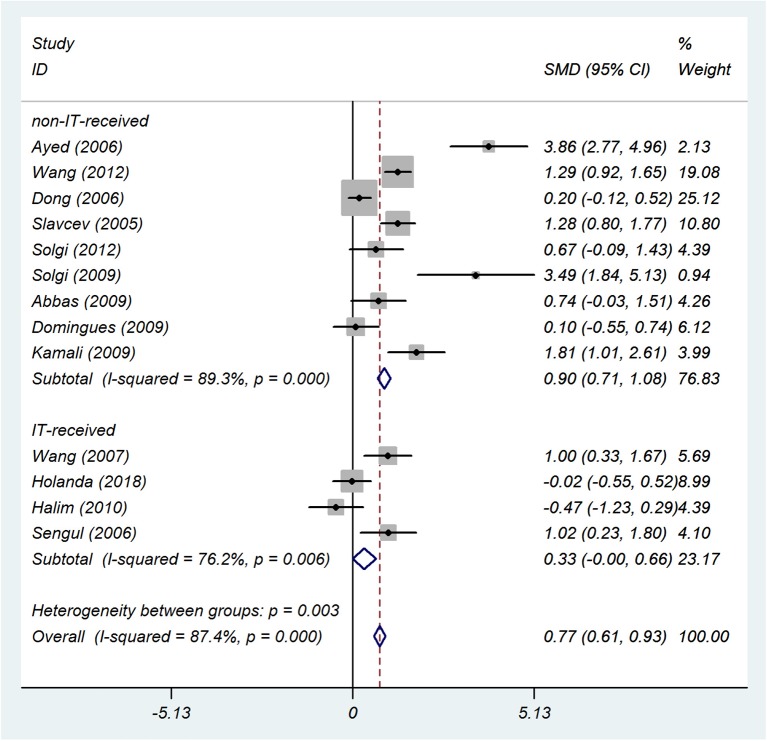
IT subgroup analysis. The forest plot shows the association between sCD30 and acute rejection during the second week after transplantation regarding the IT subgroup. SMD, Standardized mean difference; CI, Confidence interval.

Among 13 studies, four studies used IT. For studies that used IT, the results were SMD 0.33; 95% CI 0–0.66; *I*^2^ = 76%, and for studies that did not use IT, the results were SMD 0.9; 95% CI 0.71–1.08; *I*^2^ = 89% (Figure 10).

##### Methodological quality of studies

Of 13 studies, 12 studies had high methodological quality and displayed a strong association between sCD30 and acute rejection (SMD 0.74; 95% CI 0.58–0.90; *I*^2^ = 87%). The results of one study of low methodological quality were SMD 3.49; CI 1.84–5.13, which were considered to be inconclusive results (Supplementary Figure 8).

##### Panel reactive antibody

Of 13 studies, the data of PRA were observed in 12 studies. The studies with a PRA <10% and with a PRA >10% demonstrated a strong and moderate association between sCD30 and acute rejection (SMD 1.14; 95% CI 0.88–1.40; *I*^2^ = 55.3% vs. SMD 0.41; 95% CI 0.21–0.62; *I*^2^ = 85.4%, respectively) (Supplementary Figure 9).

Meta-regression was performed on the age and CIT of patients with rejection. The age of patients with rejection showed no relation to the strong effect of sCD30 (Supplementary Figure 10). However, meta-regression on CIT indicated a positive correlation between the high time of CIT and the stronger effect of sCD30 (Supplementary Figure 11).

#### The 3 Week-Post-transplant sCD30 Level and Acute Rejection Status

Three studies had data of the 3 week-post-transplant sCD30 level and acute rejection status. The results showed a strong association between sCD30 and acute rejection (SMD 1.11; 95% CI 0.67–1.56; *I*^2^ = 94%) (Supplementary Figure 12), which is consistent with the results of the 1 and 2 week-post-transplant sCD30.

#### The 4 Week and 1 Month-Post-transplant sCD30 Level and Acute Rejection Status

Three and five studies had data of 4 week- and 1 month-post-transplant sCD30, respectively, and both of them indicated that serum level of sCD30 had strong association with acute rejection (SMD 1.27; 95% CI 0.78–1.76; *I*^2^ = 97.2% vs. SMD 0.71; 95% CI 0.34–1.07; *I*^2^ = 88.4%, respectively) (Supplementary Figures 13, 14).

## Discussion

In general, a total of 1,453 patients in 18 studies were included in this paper. This is the first systematic review and meta-analysis addressed the association of post-transplant sCD30 with acute rejection during different periods after transplantation. With regard to the different measurement times of sCD30 among the five groups after transplantation, and the main data for meta-analysis were divided into five sections (see Study Characteristics section). In addition, we analyzed one more group within the 1 week-post-transplant sCD30 in which sCD30 was measured before or at the time of acute rejection.

In this study, all the included studies tried to assess whether post-transplant sCD30 was associated with or was a risk factor for acute rejection. Because of a shared methodology (such as the study type, the same manner of sCD30 measurement, and the same immunosuppressive therapy), they could be considered as one study with a large sample size. In this regard, they were analyzed with a fixed-effect model. The study subjects of included studies had different characteristics, such as different donor types, PRA, gender, CIT, etc. (see Table 1 and Supplementary Table 7). The data from each study could include these different characteristics. The studies had inconsistent results regarding the role of sCD30 as a risk factor for acute rejection. This is because of the different characteristics of study subjects (which are discussed in the next sixth paragraph).

In this meta-analysis, we observed that there is a strong association between the post-transplant sCD30 level and acute rejection in kidney transplant recipients. In other words, there was a strong association between sCD30 and acute rejection in all different measurement times after the transplantation (SMD 1.22, 0.77, 1.11, 1.27, and 0.71). These results were obtained without considerable publication bias, which was proved by the Trim and Fill method.

Moreover, we found that patients with acute rejection had a significantly higher post-transplant sCD30 level than the patients without the rejection in which post-transplant sCD30 was measured before or at the time of the rejection. In other words, there was a strong association between the post-transplant sCD30 and the risk of acute rejection.

As a result of alloimmune stimulation, CD30^+^ T cells could be generated, and they shed CD30 to the blood, which implies cellular immune stimulation against the allograft. Furthermore, CD30^+^ T cells produce a high amount of INF-γ and IL-5, which are associated with early graft rejection (40, 41). A high amount of sCD30 in the blood of transplant recipients before acute rejection could imply activation of T cells against the allograft and could lead to cytokine production. This activation, in combination with pre-transplant antibodies, could finally lead to allograft rejection (14). In general, measurement of sCD30 early after the transplantation may be helpful and give valuable information about the allograft status.

Subgroup analyses (the number of transplanted kidneys, donor type, methodological quality, and PRA) showed consistent results with the results of both 1 and 2 week post-transplant sCD30 levels. However, the subgroups with ≤3 studies were considered to result in an inconclusive result.

Here, we evaluated each subgroup analysis on the basis of their effect on heterogeneity and pooled estimate. We assessed the subgroup analyses in the 1 week-post-transplant sCD30. The methodological quality and PRA did not justify the heterogeneity and did not change the pooled estimate. The number of transplanted kidneys subgroup did not justify heterogeneity, but the pooled estimate dropped from 1.22 to 0.74.

The donor type of subgroup analysis interestingly justified the severe heterogeneity. The heterogeneity dropped from 94.2 to 0.0% in the living type and 94.2 to 60% in the deceased type. Thus, the most critical factor, caused to severe heterogeneity, was donor type. It implies the effect of donor type in heterogeneous results of primary studies.

Moreover, this subgroup analysis showed that the pooled estimate had a considerable change in the living type, which decreased from 1.22 to 0.49. Nevertheless, there was a moderate association between sCD30 and acute rejection. As it was expectable, in the deceased type, the increased pooled estimate was observed (from 1.22 to 2.56). Although a strong association was found between sCD30 and acute rejection, this effect was moderate in the patients who received the graft from living donors. It means that the donor type, along with sCD30, increased the risk of acute rejection. In general, it was clarified that the donor type is one of the major causes of heterogeneous results in primary studies.

Immunosuppressive drugs may affect the sCD30 level and decrease the post-transplant sCD30 level. However, patients with acute rejection have a higher sCD30 level than those without rejection (30). Some of the studies reported that the sCD30 level was not significantly different between the two distinct immunosuppressive regimens (12, 28, 29, 37). Moreover, one of the studies reported that the sCD30 level was not different between the IT-received group and the non-IT-received group (37).

However, our subgroup analyses (both in the 1 and 2 week groups) showed that the sCD30 level was higher in the non-IT-received group than the IT-received group; moreover, the effect size increased from 1.22 to 1.31 in the 1-week group and from 0.77 to 0.90 in the 2 week group. In the IT-received group, the effect size decreased from 1.22 to 0.60 in the 1 week group and from 0.77 to 0.33 in the 2 week group. This means that there was a moderate association between the sCD30 level and the acute rejection in the patients who received IT. Moreover, it could be implied that IT decreased the sCD30 level in the patients with acute rejection, and this may help the immunoregulatory function of CD30^+^ T cells (see the seventh paragraph of Introduction section). If the IT data of included studies reported separately in the patients with and without rejection, the reliability of this result would be significantly improved.

Meta-regression analysis showed no relation between the effect of sCD30 and the age of patients with rejection. However, there was a positive correlation between increased CIT and sCD30 effect; thus, the increase of CIT led to an increase of the sCD30 effect. The increase of CIT, along with donor type and sCD30, is therefore another factor that results in a higher rate of acute rejection. The sensitivity analysis also did not change the pooled estimate when the studies were separately omitted.

The results of the subgroup analysis on the 2 week-post-transplant sCD30 were that the methodological quality did not justify the heterogeneity and did not alter the pooled estimate. However, in the number of transplanted kidneys subgroup, the one-transplant-recipient subgroup increased the effect size from 0.77 to 1.35, but heterogeneity was not justified. Although in the several-transplant-recipient subgroup, the heterogeneity and effect size were decreased, the results were considered to be inconclusive because of two studies.

In the donor type subgroup, heterogeneity showed no acceptable decrease, but the pooled estimate in the living subgroup decreased from 0.77 to 0.5, and in the deceased subgroup it increased from 0.77 to 0.81. This indicates that sCD30 in the patients who received the graft from living donors was of a moderate relation to acute rejection. In contrast, sCD30 in the patients who received the graft from a deceased donor was of a strong relation to acute rejection.

In the PRA subgroup analysis, the PRA >10% reduced the heterogeneity from 87.4 to 55.3% and increased the pooled estimate from 0.77 to 1.14. However, in the PRA <10%, the heterogeneity did not decrease, but the pooled estimate dropped from 0.77–0.41.

The sCD30 is considered to be a marker for T-cell activation and response in which T cells can damage the graft. In the two different studies from 2016, Süsal and colleagues indicated that if donor-specific antibodies ([DSA] pre-existing or *de novo*) receive T-cell help, they will contribute to ABMR. Thus, an increased sCD30 level, which indicated T-cell activation and help, was considered to be an important marker that, in combination with DSA, resulted in ABMR and graft loss. Interestingly, pre-transplant DSA was only related to ABMR in patients with high pre-transplant sCD30 level implying an essential role of pre-transplant sCD30 in the graft loss. In other words, pre-transplant DSA did not culminate in ABMR in the patients with a low level of sCD30.

Consistently, the present meta-analysis indicated an inevitable role of sCD30 (post-transplant) in the prediction of acute rejection. The Süsal studies and the present meta-analysis indicated that both pre-transplant and post-transplant sCD30 levels have an indispensable role in kidney rejection. Therefore, monitoring sCD30 after transplantation could be used as a predictive biomarker and help to prevent rejection. This monitoring in some patients (who received a graft from a deceased donor, who did not receive IT, and who had high CIT) could be much more important. Here, we could suggest that the clinicians can consider the early-post-transplant sCD30 measurement of kidney transplant recipients for the better management and if possible to prevent the probable acute rejection. Another suggestion is that they should reduce the CIT of the patients' graft.

We acknowledge some limitations: (i) the subgroup analyses with ≤3 studies were considered to be inconclusive results; (ii) data of the PRA could not be as reliable as other data because the PRA data were not collected separately from each group; (iii) there were not enough studies in the 3 and 4 week-post-transplant sCD30 (three studies in each group); and (iv) the exclusion of one case-control study since there were no more case-control studies to analyze together.

In conclusion, in this systematic review and meta-analysis, we indicated that post-transplant sCD30 have a strong association with acute rejection despite the severe heterogeneity. We further found that the donor type is the most important factor leading to heterogeneous results in primary studies. We also found that there are high levels of sCD30 in the donor type (deceased) and IT (non-IT-received) subgroups, as well as in the increased CIT. This implies the activation of cellular immune response and further allograft damage in these patients. Moreover, a moderate association of sCD30 with acute rejection in the living donors and IT-received group implies a remarkable role of sCD30 in the occurrence of acute rejection. In the end, a living donor, IT therapy, and low CIT could decrease the level of sCD30 and result in long-term allograft survival.

## Data Availability Statement

The datasets analyzed in this article are not publicly available. Requests to access the datasets should be directed to m.mohammadnia@mubabol.ac.ir.

## Author Contributions

MM screened and selected studies, extracted and analyzed data, and wrote the manuscript. MS screened and selected studies, extracted data, and assessed the quality of studies. RA assessed the quality of studies and wrote the manuscript. ID reviewed and revised the manuscript. EN analyzed data. MM-A designed study and reviewed and revised the final version of the manuscript.

### Conflict of Interest

The authors declare that the research was conducted in the absence of any commercial or financial relationships that could be construed as a potential conflict of interest.
