# Supplementary material for: Soluble CD30, the Immune Response, and Acute Rejection in Human Kidney Transplantation: A Systematic Review and Meta-Analysis
Source: Front Immunol. 2020 Feb 28;11:295. doi: 10.3389/fimmu.2020.00295 (PMC7093023; doi:10.3389/fimmu.2020.00295)
Supplement: Supplementary file 2 [file Table_2.docx]

| Search round | Syntax in PubMed | NNR | Output No. | Search Day |
| --- | --- | --- | --- | --- |
| 1 | ("Ki-1 Antigen" OR (Antigen AND Ki-1) OR "Ki 1 Antigen" OR "CD30 Antigens" OR (Antigens AND CD30) OR "Ber-H2 Antigen" OR (Antigen AND Ber-H2) OR "Ber H2 Antigen" OR "TNFRSF8 Receptor" OR (Receptor AND TNFRSF8) OR (Antigens AND Ki-1) OR (Antigens AND "Ki 1") OR "Ki-1 Antigens" OR "Ki 1 Antigens" OR ("Tumor Necrosis Factor Receptor Superfamily" AND "Member 8") OR "CD30 Antigen" OR (Antigen AND CD30) OR "Ber-H2 Antigens" OR (Antigens AND Ber-H2) OR "Ber H2 Antigens" OR "tumor necrosis factor" OR Ber-H2 OR CD30L OR CD30 OR TNFRSF8 OR "Soluble CD30" OR sCD30) **AND** ("kidney Transplantation" OR "renal transplantation" OR "graft rejection" OR "acute graft rejection" OR (transplantation AND kidney) OR (transplantation AND renal) OR allotransplantation OR "acute allograft rejection" OR "kidney graft rejection" OR "renal graft rejection" OR "acute homograft rejection" OR "cell-mediated rejection" OR "antibody-mediated rejection" OR ("graft rejection" AND acute)) **AND** 1990/01/01:2018/04/30[dp] | ~34 | 1909 | 2018/05/31 |
| 2 | ("Ki-1 Antigen"[tiab] OR (Antigen[tiab] AND Ki-1[tiab]) OR "Ki 1 Antigen"[tiab] OR "CD30 Antigens"[tiab] OR (Antigens[tiab] AND CD30[tiab]) OR "Ber-H2 Antigen"[tiab] OR (Antigen[tiab] AND Ber-H2[tiab]) OR "Ber H2 Antigen"[tiab] OR "TNFRSF8 Receptor"[tiab] OR (Receptor[tiab] AND TNFRSF8[tiab]) OR (Antigens[tiab] AND Ki-1[tiab]) OR (Antigens[tiab] AND "Ki 1"[tiab]) OR "Ki-1 Antigens"[tiab] OR "Ki 1 Antigens" OR ("Tumor Necrosis Factor Receptor Superfamily" AND "Member 8") OR "CD30 Antigen" OR (Antigen AND CD30) OR "Ber-H2 Antigens" OR (Antigens AND Ber-H2) OR "Ber H2 Antigens" OR "tumor necrosis factor" OR Ber-H2 OR CD30L OR CD30 OR TNFRSF8 OR "Soluble CD30" OR sCD30) AND ("kidney Transplantation"[tiab] OR "renal transplantation"[tiab] OR "graft rejection"[tiab] OR "acute graft rejection" OR (transplantation AND kidney) OR (transplantation AND renal) OR allotransplantation OR "acute allograft rejection" OR "kidney graft rejection" OR "renal graft rejection" OR "acute homograft rejection" OR "cell-mediated rejection" OR "antibody-mediated rejection" OR ("graft rejection" AND acute)) AND 1990/01/01:2018/04/30[dp] | ~ 34 | 1550 |  |
| 3 | ("Ki-1 Antigen"[tiab] OR (Antigen[tiab] AND Ki-1[tiab]) OR "Ki 1 Antigen"[tiab] OR "CD30 Antigens"[tiab] OR (Antigens[tiab] AND CD30[tiab]) OR "Ber-H2 Antigen"[tiab] OR (Antigen[tiab] AND Ber-H2[tiab]) OR "Ber H2 Antigen"[tiab] OR "TNFRSF8 Receptor"[tiab] OR (Receptor[tiab] AND TNFRSF8[tiab]) OR (Antigens[tiab] AND Ki-1[tiab]) OR (Antigens[tiab] AND "Ki 1"[tiab]) OR "Ki-1 Antigens"[tiab] OR "Ki 1 Antigens"[tiab] OR ("Tumor Necrosis Factor Receptor Superfamily"[tiab] AND "Member 8"[tiab]) OR "CD30 Antigen"[tiab] OR (Antigen AND CD30) OR "Ber-H2 Antigens" OR (Antigens AND Ber-H2) OR "Ber H2 Antigens" OR "tumor necrosis factor" OR Ber-H2 OR CD30L OR CD30 OR TNFRSF8 OR "Soluble CD30" OR sCD30) AND ("kidney Transplantation"[tiab] OR "renal transplantation"[tiab] OR "graft rejection"[tiab] OR "acute graft rejection"[tiab] OR (transplantation[tiab] AND kidney[tiab]) OR (transplantation[tiab] AND renal[tiab]) OR allotransplantation OR "acute allograft rejection" OR "kidney graft rejection" OR "renal graft rejection" OR "acute homograft rejection" OR "cell-mediated rejection" OR "antibody-mediated rejection" OR ("graft rejection" AND acute)) AND 1990/01/01:2018/04/30[dp] | 25 | 1073 |  |
| 4 | ("Ki-1 Antigen"[tiab] OR (Antigen[tiab] AND Ki-1[tiab]) OR "Ki 1 Antigen"[tiab] OR "CD30 Antigens"[tiab] OR (Antigens[tiab] AND CD30[tiab]) OR "Ber-H2 Antigen"[tiab] OR (Antigen[tiab] AND Ber-H2[tiab]) OR "Ber H2 Antigen"[tiab] OR "TNFRSF8 Receptor"[tiab] OR (Receptor[tiab] AND TNFRSF8[tiab]) OR (Antigens[tiab] AND Ki-1[tiab]) OR (Antigens[tiab] AND "Ki 1"[tiab]) OR "Ki-1 Antigens"[tiab] OR "Ki 1 Antigens"[tiab] OR ("Tumor Necrosis Factor Receptor Superfamily"[tiab] AND "Member 8"[tiab]) OR "CD30 Antigen"[tiab] OR (Antigen[tiab] AND CD30[tiab]) OR "Ber-H2 Antigens"[tiab] OR (Antigens[tiab] AND Ber-H2[tiab]) OR "Ber H2 Antigens"[tiab] OR "tumor necrosis factor"[tiab] OR Ber-H2 OR CD30L OR CD30 OR TNFRSF8 OR "Soluble CD30" OR sCD30) AND ("kidney Transplantation"[tiab] OR "renal transplantation"[tiab] OR "graft rejection"[tiab] OR "acute graft rejection"[tiab] OR (transplantation[tiab] AND kidney[tiab]) OR (transplantation[tiab] AND renal[tiab]) OR allotransplantation[tiab] OR "acute allograft rejection"[tiab] OR "kidney graft rejection"[tiab] OR "renal graft rejection"[tiab] OR "acute homograft rejection"[tiab] OR "cell-mediated rejection" OR "antibody-mediated rejection" OR ("graft rejection" AND acute)) AND 1990/01/01:2018/04/30[dp] | ~ 14 | 715 |  |

Table S2. Search strategy in PubMed.
