# Supplementary material for: Soluble CD30, the Immune Response, and Acute Rejection in Human Kidney Transplantation: A Systematic Review and Meta-Analysis
Source: Front Immunol. 2020 Feb 28;11:295. doi: 10.3389/fimmu.2020.00295 (PMC7093023; doi:10.3389/fimmu.2020.00295)
Supplement: Supplementary file 3 [file Table_3.docx]

| Search round | Syntax in scopus | NNR | Output No. | Search Day |
| --- | --- | --- | --- | --- |
| 1 | (TITLE-ABS("Ki-1 Antigen") OR (TITLE-ABS(Antigen) AND TITLE-ABS(Ki-1)) OR TITLE-ABS("Ki 1 Antigen") OR TITLE-ABS("CD30 Antigens") OR (TITLE-ABS(Antigens) AND TITLE-ABS(CD30)) OR TITLE-ABS("Ber-H2 Antigen") OR (TITLE-ABS(Antigen) AND TITLE-ABS(Ber-H2)) OR TITLE-ABS("Ber H2 Antigen") OR TITLE-ABS("TNFRSF8 Receptor") OR (TITLE-ABS(Receptor) AND TITLE-ABS(TNFRSF8)) OR (TITLE-ABS(Antigens) AND TITLE-ABS(Ki-1)) OR (TITLE-ABS(Antigens) AND TITLE-ABS("Ki 1")) OR TITLE-ABS("Ki-1 Antigens") OR TITLE-ABS("Ki 1 Antigens") OR (TITLE-ABS("Tumor Necrosis Factor Receptor Superfamily") AND TITLE-ABS("Member 8")) OR TITLE-ABS("CD30 Antigen") OR (TITLE-ABS(Antigen) AND TITLE-ABS(CD30)) OR TITLE-ABS("Ber-H2 Antigens") OR (TITLE-ABS(Antigens) AND TITLE-ABS(Ber-H2)) OR TITLE-ABS("Ber H2 Antigens") OR TITLE-ABS("tumor necrosis factor") OR ALL(Ber-H2) OR ALL(CD30L) OR ALL(CD30) OR ALL(TNFRSF8) OR ALL("Soluble CD30") OR ALL(sCD30)) AND (TITLE-ABS("kidney Transplantation") OR TITLE-ABS("renal transplantation") OR TITLE-ABS("graft rejection") OR TITLE-ABS("acute graft rejection") OR (TITLE-ABS(transplantation) AND TITLE-ABS(kidney)) OR (TITLE-ABS(transplantation) AND TITLE-ABS(renal)) OR TITLE-ABS(allotransplantation) OR TITLE-ABS("acute allograft rejection") OR TITLE-ABS("kidney graft rejection") OR TITLE-ABS("renal graft rejection") OR TITLE-ABS("acute homograft rejection") OR ALL("cell-mediated rejection") OR ALL("antibody-mediated rejection") OR (ALL("graft rejection") AND ALL(acute))) AND (PUBYEAR > 1990 AND PUBYEAR < 2018) OR PUBDATETXT(April 2018) | 50 | 2040 | 2018/06/02 |
| 2 | (TITLE-ABS("Ki-1 Antigen") OR (TITLE-ABS(Antigen) AND TITLE-ABS(Ki-1)) OR TITLE-ABS("Ki 1 Antigen") OR TITLE-ABS("CD30 Antigens") OR (TITLE-ABS(Antigens) AND TITLE-ABS(CD30)) OR TITLE-ABS("Ber-H2 Antigen") OR (TITLE-ABS(Antigen) AND TITLE-ABS(Ber-H2)) OR TITLE-ABS("Ber H2 Antigen") OR TITLE-ABS("TNFRSF8 Receptor") OR (TITLE-ABS(Receptor) AND TITLE-ABS(TNFRSF8)) OR (TITLE-ABS(Antigens) AND TITLE-ABS(Ki-1)) OR (TITLE-ABS(Antigens) AND TITLE-ABS("Ki 1")) OR TITLE-ABS("Ki-1 Antigens") OR TITLE-ABS("Ki 1 Antigens") OR (TITLE-ABS("Tumor Necrosis Factor Receptor Superfamily") AND TITLE-ABS("Member 8")) OR TITLE-ABS("CD30 Antigen") OR (TITLE-ABS(Antigen) AND TITLE-ABS(CD30)) OR TITLE-ABS("Ber-H2 Antigens") OR (TITLE-ABS(Antigens) AND TITLE-ABS(Ber-H2)) OR TITLE-ABS("Ber H2 Antigens") OR TITLE-ABS("tumor necrosis factor") OR TITLE-ABS(Ber-H2) OR TITLE-ABS(CD30L) OR TITLE-ABS(CD30) OR ALL(TNFRSF8) OR ALL("Soluble CD30") OR ALL(sCD30)) AND (TITLE-ABS("kidney Transplantation") OR TITLE-ABS("renal transplantation") OR TITLE-ABS("graft rejection") OR TITLE-ABS("acute graft rejection") OR (TITLE-ABS(transplantation) AND TITLE-ABS(kidney)) OR (TITLE-ABS(transplantation) AND TITLE-ABS(renal)) OR TITLE-ABS(allotransplantation) OR TITLE-ABS("acute allograft rejection") OR TITLE-ABS("kidney graft rejection") OR TITLE-ABS("renal graft rejection") OR TITLE-ABS("acute homograft rejection") OR TITLE-ABS("cell-mediated rejection") OR TITLE-ABS("antibody-mediated rejection") OR (ALL("graft rejection") AND ALL(acute))) AND (PUBYEAR > 1990 AND PUBYEAR < 2018) OR PUBDATETXT(April 2018) | 50 | 1647 | 2018/06/02 |
| 3 | (TITLE-ABS("Ki-1 Antigen") OR (TITLE-ABS(Antigen) AND TITLE-ABS(Ki-1)) OR TITLE-ABS("Ki 1 Antigen") OR TITLE-ABS("CD30 Antigens") OR (TITLE-ABS(Antigens) AND TITLE-ABS(CD30)) OR TITLE-ABS("Ber-H2 Antigen") OR (TITLE-ABS(Antigen) AND TITLE-ABS(Ber-H2)) OR TITLE-ABS("Ber H2 Antigen") OR TITLE-ABS("TNFRSF8 Receptor") OR (TITLE-ABS(Receptor) AND TITLE-ABS(TNFRSF8)) OR (TITLE-ABS(Antigens) AND TITLE-ABS(Ki-1)) OR (TITLE-ABS(Antigens) AND TITLE-ABS("Ki 1")) OR TITLE-ABS("Ki-1 Antigens") OR TITLE-ABS("Ki 1 Antigens") OR (TITLE-ABS("Tumor Necrosis Factor Receptor Superfamily") AND TITLE-ABS("Member 8")) OR TITLE-ABS("CD30 Antigen") OR (TITLE-ABS(Antigen) AND TITLE-ABS(CD30)) OR TITLE-ABS("Ber-H2 Antigens") OR (TITLE-ABS(Antigens) AND TITLE-ABS(Ber-H2)) OR TITLE-ABS("Ber H2 Antigens") OR TITLE-ABS("tumor necrosis factor") OR TITLE-ABS(Ber-H2) OR TITLE-ABS(CD30L) OR TITLE-ABS(CD30) OR TITLE-ABS(TNFRSF8) OR TITLE-ABS("Soluble CD30") OR TITLE-ABS(sCD30)) AND (TITLE-ABS("kidney Transplantation") OR TITLE-ABS("renal transplantation") OR TITLE-ABS("graft rejection") OR TITLE-ABS("acute graft rejection") OR (TITLE-ABS(transplantation) AND TITLE-ABS(kidney)) OR (TITLE-ABS(transplantation) AND TITLE-ABS(renal)) OR TITLE-ABS(allotransplantation) OR TITLE-ABS("acute allograft rejection") OR TITLE-ABS("kidney graft rejection") OR TITLE-ABS("renal graft rejection") OR TITLE-ABS("acute homograft rejection") OR TITLE-ABS("cell-mediated rejection") OR TITLE-ABS("antibody-mediated rejection") OR (ALL("graft rejection") AND ALL(acute))) AND (PUBYEAR > 1990 AND PUBYEAR < 2018) OR PUBDATETXT(April 2018) | ~ 33 | 1405 | 2018/06/02 |

Table S3. Search strategy in Scopus.
