# Supplementary material for: Soluble CD30, the Immune Response, and Acute Rejection in Human Kidney Transplantation: A Systematic Review and Meta-Analysis
Source: Front Immunol. 2020 Feb 28;11:295. doi: 10.3389/fimmu.2020.00295 (PMC7093023; doi:10.3389/fimmu.2020.00295)
Supplement: Supplementary file 4 [file Table_4.docx]

| Search round | Syntax WOS | NNR | Output No. | Search Day |
| --- | --- | --- | --- | --- |
| 1 | (TS=("Ki-1 Antigen") OR (TS=(Antigen) AND TS=(Ki-1)) OR TS=("Ki 1 Antigen") OR TS=("CD30 Antigens") OR (TS=(Antigens) AND TS=(CD30)) OR TS=("Ber-H2 Antigen") OR (TS=(Antigen) AND TS=(Ber-H2)) OR TS=("Ber H2 Antigen") OR TS=("TNFRSF8 Receptor") OR (TS=(Receptor) AND TS=(TNFRSF8)) OR (TS=(Antigens) AND TS=(Ki-1)) OR (TS=(Antigens) AND TS=("Ki 1")) OR TS=("Ki-1 Antigens") OR TS=("Ki 1 Antigens") OR (TS=("Tumor Necrosis Factor Receptor Superfamily") AND TS=("Member 8")) OR TS=("CD30 Antigen") OR (TS=(Antigen) AND TS=(CD30)) OR TS=("Ber-H2 Antigens") OR (TS=(Antigens) AND TS=(Ber-H2)) OR TS=("Ber H2 Antigens") OR TS=("tumor necrosis factor") OR TS=(Ber-H2) OR TS=(CD30L) OR TS=(CD30) OR TS=(TNFRSF8) OR TS=("Soluble CD30") OR TS=(sCD30)) AND (TS=("kidney Transplantation") OR TS=("renal transplantation") OR TS=("graft rejection") OR TS=("acute graft rejection") OR (TS=(transplantation) AND TS=(kidney)) OR (TS=(transplantation) AND TS=(renal)) OR TS=(allotransplantation) OR TS=("acute allograft rejection") OR TS=("kidney graft rejection") OR TS=("renal graft rejection") OR TS=("acute homograft rejection") OR TS=("cell-mediated rejection") OR TS=("antibody-mediated rejection") OR (TS=("graft rejection") AND TS=(acute))) AND PY=(1990-2018) | 25 | 1270 | 2018/06/04 |

Table S4. Search strategy in Web of Sciences.
