# Supplementary material for: Soluble CD30, the Immune Response, and Acute Rejection in Human Kidney Transplantation: A Systematic Review and Meta-Analysis
Source: Front Immunol. 2020 Feb 28;11:295. doi: 10.3389/fimmu.2020.00295 (PMC7093023; doi:10.3389/fimmu.2020.00295)
Supplement: Supplementary file 5 [file Table_5.docx]

| Search round | Syntax in ProQuest | NNR | Output No. | Search Day |
| --- | --- | --- | --- | --- |
| 1 | (AB,TI("Ki-1 Antigen") OR (AB,TI(Antigen) AND AB,TI(Ki-1)) OR AB,TI("Ki 1 Antigen") OR AB,TI("CD30 Antigens") OR (AB,TI(Antigens) AND AB,TI(CD30)) OR AB,TI("Ber-H2 Antigen") OR (AB,TI(Antigen) AND AB,TI(Ber-H2)) OR AB,TI("Ber H2 Antigen") OR AB,TI("TNFRSF8 Receptor") OR (AB,TI(Receptor) AND AB,TI(TNFRSF8)) OR (AB,TI(Antigens) AND AB,TI(Ki-1)) OR (AB,TI(Antigens) AND AB,TI("Ki 1")) OR AB,TI("Ki-1 Antigens") OR AB,TI("Ki 1 Antigens") OR (AB,TI("Tumor Necrosis Factor Receptor Superfamily") AND AB,TI("Member 8")) OR AB,TI("CD30 Antigen") OR (AB,TI(Antigen) AND AB,TI(CD30)) OR AB,TI("Ber-H2 Antigens") OR (AB,TI(Antigens) AND AB,TI(Ber-H2)) OR AB,TI("Ber H2 Antigens") OR AB,TI("tumor necrosis factor") OR AB,TI(Ber-H2) OR AB,TI(CD30L) OR AB,TI(CD30) OR AB,TI(TNFRSF8) OR AB,TI("Soluble CD30") OR AB,TI(sCD30)) AND (AB,TI("kidney Transplantation") OR AB,TI("renal transplantation") OR AB,TI("graft rejection") OR AB,TI("acute graft rejection") OR (AB,TI(transplantation) AND AB,TI(kidney)) OR (AB,TI(transplantation) AND AB,TI(renal)) OR AB,TI(allotransplantation) OR AB,TI("acute allograft rejection") OR AB,TI("kidney graft rejection") OR AB,TI("renal graft rejection") OR AB,TI("acute homograft rejection") OR AB,TI("cell-mediated rejection") OR AB,TI("antibody-mediated rejection") OR (ALL,FT("graft rejection") AND ALL,FT(acute))) | 50 | 1396 | 2018/06/04 |
| 2 | (AB,TI("Ki-1 Antigen") OR (AB,TI(Antigen) AND AB,TI(Ki-1)) OR AB,TI("Ki 1 Antigen") OR AB,TI("CD30 Antigens") OR (AB,TI(Antigens) AND AB,TI(CD30)) OR AB,TI("Ber-H2 Antigen") OR (AB,TI(Antigen) AND AB,TI(Ber-H2)) OR AB,TI("Ber H2 Antigen") OR AB,TI("TNFRSF8 Receptor") OR (AB,TI(Receptor) AND AB,TI(TNFRSF8)) OR (AB,TI(Antigens) AND AB,TI(Ki-1)) OR (AB,TI(Antigens) AND AB,TI("Ki 1")) OR AB,TI("Ki-1 Antigens") OR AB,TI("Ki 1 Antigens") OR (AB,TI("Tumor Necrosis Factor Receptor Superfamily") AND AB,TI("Member 8")) OR AB,TI("CD30 Antigen") OR (AB,TI(Antigen) AND AB,TI(CD30)) OR AB,TI("Ber-H2 Antigens") OR (AB,TI(Antigens) AND AB,TI(Ber-H2)) OR AB,TI("Ber H2 Antigens") OR AB,TI("tumor necrosis factor") OR AB,TI(Ber-H2) OR AB,TI(CD30L) OR AB,TI(CD30) OR AB,TI(TNFRSF8) OR AB,TI("Soluble CD30") OR AB,TI(sCD30)) AND (AB,TI("kidney Transplantation") OR AB,TI("renal transplantation") OR AB,TI("graft rejection") OR AB,TI("acute graft rejection") OR (AB,TI(transplantation) AND AB,TI(kidney)) OR (AB,TI(transplantation) AND AB,TI(renal)) OR AB,TI(allotransplantation) OR AB,TI("acute allograft rejection") OR AB,TI("kidney graft rejection") OR AB,TI("renal graft rejection") OR AB,TI("acute homograft rejection") OR AB,TI("cell-mediated rejection") OR AB,TI("antibody-mediated rejection") OR (AB,TI("graft rejection") AND AB,TI(acute))) | ~ 33 | 1014 | 2018/06/04 |

Table S5. Search strategy in ProQuest.
