# Supplementary material for: Soluble CD30, the Immune Response, and Acute Rejection in Human Kidney Transplantation: A Systematic Review and Meta-Analysis
Source: Front Immunol. 2020 Feb 28;11:295. doi: 10.3389/fimmu.2020.00295 (PMC7093023; doi:10.3389/fimmu.2020.00295)
Supplement: Supplementary file 6 [file Table_6.docx]

| Search Round | Syntax in EMbase | NNR | Output No. | Search Day |
| --- | --- | --- | --- | --- |
| 1 | ("Ki-1 Antigen":ti,ab OR (Antigen:ti,ab AND Ki-1:ti,ab) OR "Ki 1 Antigen":ti,ab OR "CD30 Antigens":ti,ab OR (Antigens:ti,ab AND CD30:ti,ab) OR "Ber-H2 Antigen":ti,ab OR (Antigen:ti,ab AND Ber-H2:ti,ab) OR "Ber H2 Antigen":ti,ab OR "TNFRSF8 Receptor":ti,ab OR (Receptor:ti,ab AND TNFRSF8:ti,ab) OR (Antigens:ti,ab AND Ki-1:ti,ab) OR (Antigens:ti,ab AND "Ki 1":ti,ab) OR "Ki-1 Antigens":ti,ab OR "Ki 1 Antigens":ti,ab OR ("Tumor Necrosis Factor Receptor Superfamily":ti,ab AND "Member 8":ti,ab) OR "CD30 Antigen":ti,ab OR (Antigen:ti,ab AND CD30:ti,ab) OR "Ber-H2 Antigens":ti,ab OR (Antigens:ti,ab AND Ber-H2:ti,ab) OR "Ber H2 Antigens":ti,ab OR "tumor necrosis factor":ti,ab OR Ber-H2 OR CD30L OR CD30 OR TNFRSF8 OR "Soluble CD30" OR sCD30) AND ("kidney Transplantation":ti,ab OR "renal transplantation":ti,ab OR "graft rejection":ti,ab OR "acute graft rejection":ti,ab OR (transplantation:ti,ab AND kidney:ti,ab) OR (transplantation:ti,ab AND renal:ti,ab) OR allotransplantation:ti,ab OR "acute allograft rejection":ti,ab OR "kidney graft rejection":ti,ab OR "renal graft rejection":ti,ab OR "acute homograft rejection":ti,ab OR "cell-mediated rejection" OR "antibody-mediated rejection" OR ("graft rejection" AND acute)) AND 1990/01/01:2018/04/30[dp] |  |  |  |

Table S6. Search strategy in EMbase.
