# Supplementary material for: Soluble CD30, the Immune Response, and Acute Rejection in Human Kidney Transplantation: A Systematic Review and Meta-Analysis
Source: Front Immunol. 2020 Feb 28;11:295. doi: 10.3389/fimmu.2020.00295 (PMC7093023; doi:10.3389/fimmu.2020.00295)
Supplement: Supplementary file 7 [file Table_7.docx]

Table S7. Detailed characteristics of the 18 included studies.

| Author | country | Rejection  age | Rejection  CIT | Induction  therapy | Rejection patient No.  2-week | Rejection patient mean.  2-week | Rejection patient SD.  2-week | Stable patient No.  2-week | Stable patient mean. 2-week | Stable patient SD.  2-week |
| --- | --- | --- | --- | --- | --- | --- | --- | --- | --- | --- |
| Ayed et al.  (2006) | Tunisia | . | . | None | 18 | 210.25 | 66.5 | 34 | 59.37 | 39.06 |
| Wang et al.  (2012) | China | . | . | None | 39 | 20.2 | 12.2 | 202 | 13.5 | 5.2 |
| Hamer et al.  (2010) | UK | 38 | . | Yes | . | . | . | . | . | . |
| Wang et al.  (2007) | China | 39 | 9.1 | Yes | 11 | 18 | 10 | 59 | 8 | 10 |
| Dong et al.  (2006) | China | 38 | 8 | None | 49 | 10 | 6 | 171 | 9 | 5 |
| Slavcev et al.  (2005) | Czech Republic | 46 | 18 | None | 24 | 62.9 | 60.1 | 93 | 31.2 | 24.7 |
| Solgi et al.  (2012) | Iran | . | . | None | 9 | 46.6 | 31.8 | 31 | 17.8 | 42.87 |
| Holanda et al.  (2018) | Brazil | . | . | Yes | 18 | 58.1 | 56.6 | 55 | 58.9 | 53.2 |
| Halim et al.  (2010) | Kuwait | 45 | 2.9 | Yes | 9 | 22.5 | 8 | 28 | 33.7 | 24 |
| Solgi et al.  (2009) | Iran | . | . | None | 6 | 37.5 | 43.8 | 14 | 11.7 | 7.4 |
| Yang et al.  (2008) | China | . | . | None | . | . | . | . | . | . |
| Abbas et al.  (2009) | Pakistan | . | . | Yes | 8 | 37.75 | 40.9 | 42 | 22.04 | 21.25 |
| Trailin et al.  (2017) | Ukraine | . | . | None | . | . | . | . | . | . |
| Domingues et al. (2009) | Brazil | 34 | . | None | 13 | 71.25 | 30 | 32 | 63 | 83 |
| Kamali et al.  (2009) | Iran | 39.8 | . | None | 8 | 28.3 | 5.2 | 65 | 19.8 | 4.7 |
| Sengul et al.  (2006) | Turkey | 34 | 14 | Yes | 8 | 53.3 | 33.1 | 42 | 35.5 | 17.5 |
| Nafar et al.  (2009) | Iran | . | . | None | . | . | . | . | . | . |
| Azarpira et al.  (2010) | Iran | 32.69 | 10.5 | None | . | . | . | . | . | . |

| Author | Rejection patient No.  3-week | Rejection patient mean.  3-week | Rejection patient SD.  3-week | Stable patient No.  3-week | Stable patient mean. 3-week | Stable patient SD.  3-week |
| --- | --- | --- | --- | --- | --- | --- |
| Wang et al.  (2007) | 11 | 9 | 7 | 59 | 6 | 1 |
| Halim et al.  (2010) | 9 | 19.2 | 7 | 28 | 22.3 | 10 |
| Domingues et al. (2009) | 15 | 62.75 | 33.25 | 23 | 42 | 20 |

| Author | Rejection patient No.  4-week | Rejection patient mean.  4-week | Rejection patient SD.  4-week | Stable patient No.  4-week | Stable patient mean. 4-week | Stable patient SD.  4-week |
| --- | --- | --- | --- | --- | --- | --- |
| Hamer et al. (2010) | 14 | 71.2 | 24.8 | 18 | 29.9 | 8.3 |
| Holanda et al. (2018) | 18 | 33.3 | 29 | 55 | 26.5 | 18.8 |
| Yang et al.  (2008) | 20 | 28.16 | 25.5 | 38 | 13.5 | 2.12 |

| Author | Rejection patient No.  1-week | Rejection patient mean.  1-week | Rejection patient SD.  1-week | Stable patient No.  1-week | Stable patient mean. 1-week | Stable patient SD.  1-week |
| --- | --- | --- | --- | --- | --- | --- |
| Wang et al.  (2007) | 11 | 8 | 3 | 59 | 6 | 1 |
| Solgi et al.  (2012) | 9 | 39.01 | 44.4 | 31 | 17.3 | 23.38 |
| Halim et al.  (2010) | 9 | 15.2 | 5 | 28 | 22 | 7 |
| Solgi et al.  (2009) | 6 | 44.5 | 36.02 | 14 | 18.6 | 14 |
| Sengul et al.  (2006) | 8 | 47 | 19.6 | 42 | 42 | 24.5 |
