# Supplementary material for: Soluble CD30, the Immune Response, and Acute Rejection in Human Kidney Transplantation: A Systematic Review and Meta-Analysis
Source: Front Immunol. 2020 Feb 28;11:295. doi: 10.3389/fimmu.2020.00295 (PMC7093023; doi:10.3389/fimmu.2020.00295)
Supplement: Supplementary file 8 [file Table_8.docx]

**Table S8.** Methodological quality assessment of observational studies by New Castle-Ottawa Scale checklist.

| **Authors (years) (reference)** | **Type of study** | **NOS Classification** | | | **Total**  **score** |
| --- | --- | --- | --- | --- | --- |
|  |  | **Selection** | **Comparability** | **Outcome** |  |
| Ayed et al.  (2006) | Cohort | *** |  | *** | 6 |
| Wang et al.  (2012) | Cohort | *** | * | *** | 7 |
| Hamer et al.  (2010) | Cohort | *** | * | ** | 6 |
| Wang et al.  (2007) | Cohort | *** | * | *** | 7 |
| Dong et al.  (2006) | Cohort | **** |  | ** | 6 |
| Slavcev et al.  (2005) | Cohort | *** |  | *** | 6 |
| Solgi et al.  (2012) | Cohort | *** | * | ** | 6 |
| Holanda et al.  (2018) | Cohort | *** |  | *** | 6 |
| Halim et al.  (2010) | Cohort | *** |  | *** | 6 |
| Solgi et al.  (2009) | Cohort | *** |  | ** | 5 |
| Yang et al.  (2008) | Cohort | *** |  | * | 4 |
| Abbas et al.  (2009) | Cohort | *** |  | *** | 6 |
| Trailin et al.  (2017) | Cohort | *** | * | *** | 7 |
| Domingues et al. (2009) | Cohort | *** | * | *** | 7 |
| Kamali et al.  (2009) | Cohort | *** | * | *** | 7 |
| Sengul et al.  (2006) | Cohort | *** | * | *** | 7 |
| Nafar et al.  (2009) | Cohort | **** | * | *** | 8 |
| Azarpira et al.  (2010) | Cohort | *** | * | ** | 6 |
