# Supplementary material for: Soluble CD30, the Immune Response, and Acute Rejection in Human Kidney Transplantation: A Systematic Review and Meta-Analysis
Source: Front Immunol. 2020 Feb 28;11:295. doi: 10.3389/fimmu.2020.00295 (PMC7093023; doi:10.3389/fimmu.2020.00295)
Supplement: Supplementary file 9 [file Table_9.docx]

Table S9. Sensitivity analysis

------------------------------------------------------------------------------

Study omitted | Estimate [95% Conf. Interval]

-------------------+----------------------------------------------------------

Ayed (2006) | 1.3145369 [1.1371815 1.4918922]

Wang (2012) | .98566967 [.79992992 1.1714094]

Hamer (2010) | 1.2174466 [1.0480111 1.3868822]

Wang (2007) | 1.1346351 [.96215332 1.3071167]

Dong (2006) | .95474142 [.76935512 1.1401278]

Slavcev (2005) | 1.2174466 [1.0480111 1.3868822]

Solgi (2012) | 1.2477262 [1.0739108 1.4215417]

Holanda (2018) | 1.2174466 [1.0480111 1.3868822]

Halim (2010) | 1.2997072 [1.1258546 1.4735596]

Solgi (2009) | 1.2033558 [1.0322284 1.3744833]

Yang (2008) | 1.1446342 [.97305858 1.3162099]

Abbas (2009) | 1.243304 [1.0696274 1.4169806]

Trailin (2017) | 1.3108604 [1.1366553 1.4850655]

Domingues (2009) | 1.276701 [1.1012928 1.452109]

Kamali (2009) | 1.2174466 [1.0480111 1.3868822]

Sengul (2006) | 1.2174466 [1.0480111 1.3868822]

Nafar (2009) | 1.3558048 [1.1722865 1.5393231]

Azarpira (2010) | 1.3215085 [1.1444778 1.4985391]

-------------------+----------------------------------------------------------

Combined | 1.2174466 [1.0480111 1.3868821]

------------------------------------------------------------------------------
