# Supplementary material for: Soluble CD30, the Immune Response, and Acute Rejection in Human Kidney Transplantation: A Systematic Review and Meta-Analysis
Source: Front Immunol. 2020 Feb 28;11:295. doi: 10.3389/fimmu.2020.00295 (PMC7093023; doi:10.3389/fimmu.2020.00295)

Supplementary figure 1. Publication bias assessment


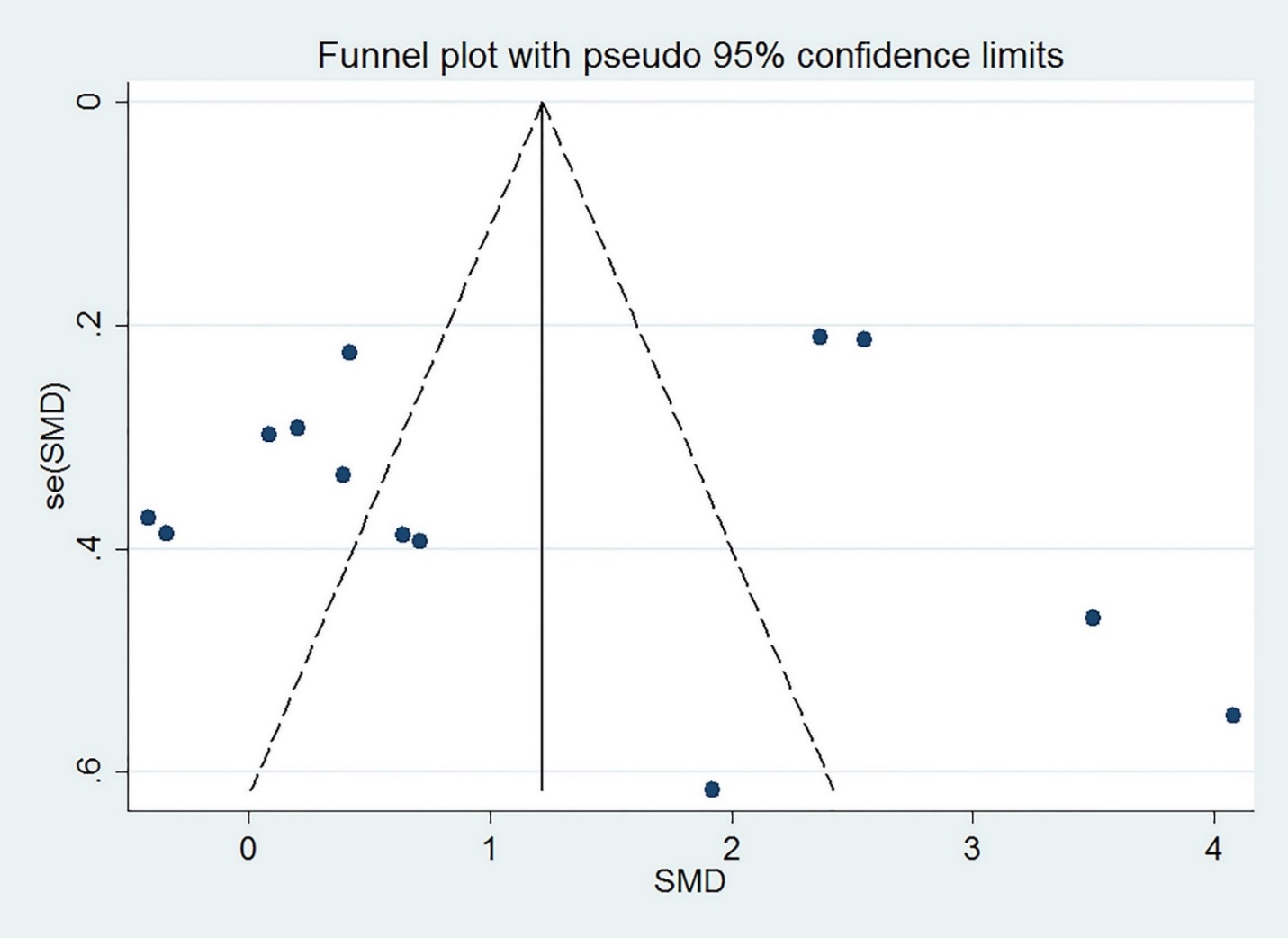


Supplementary figure 2. Subgroup by the number of transplanted kidneys in the 1-week post-transplant sCD30 group.


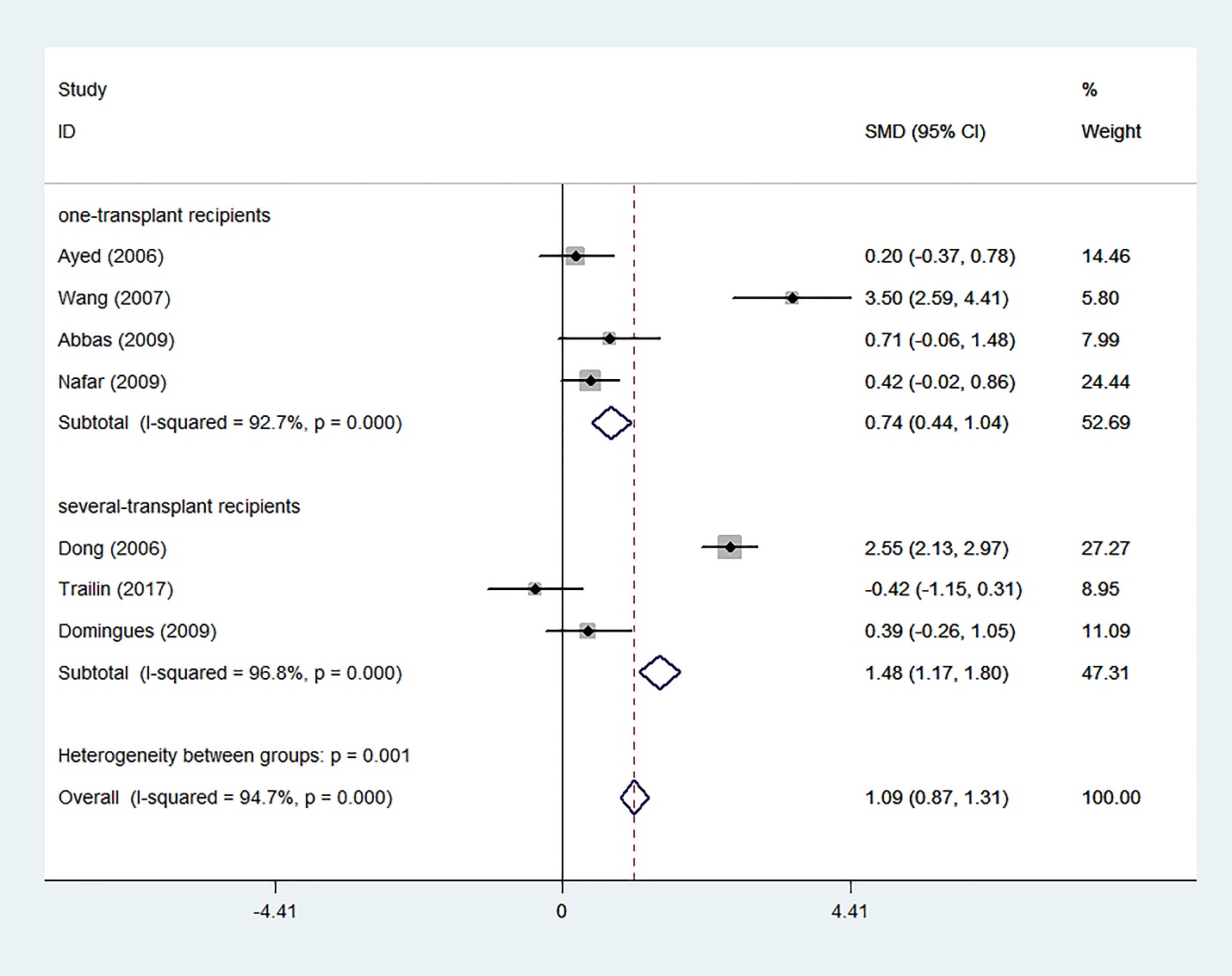


Supplementary figure 3. Subgroup by methodological quality in the 1-week post-transplant sCD30 group.


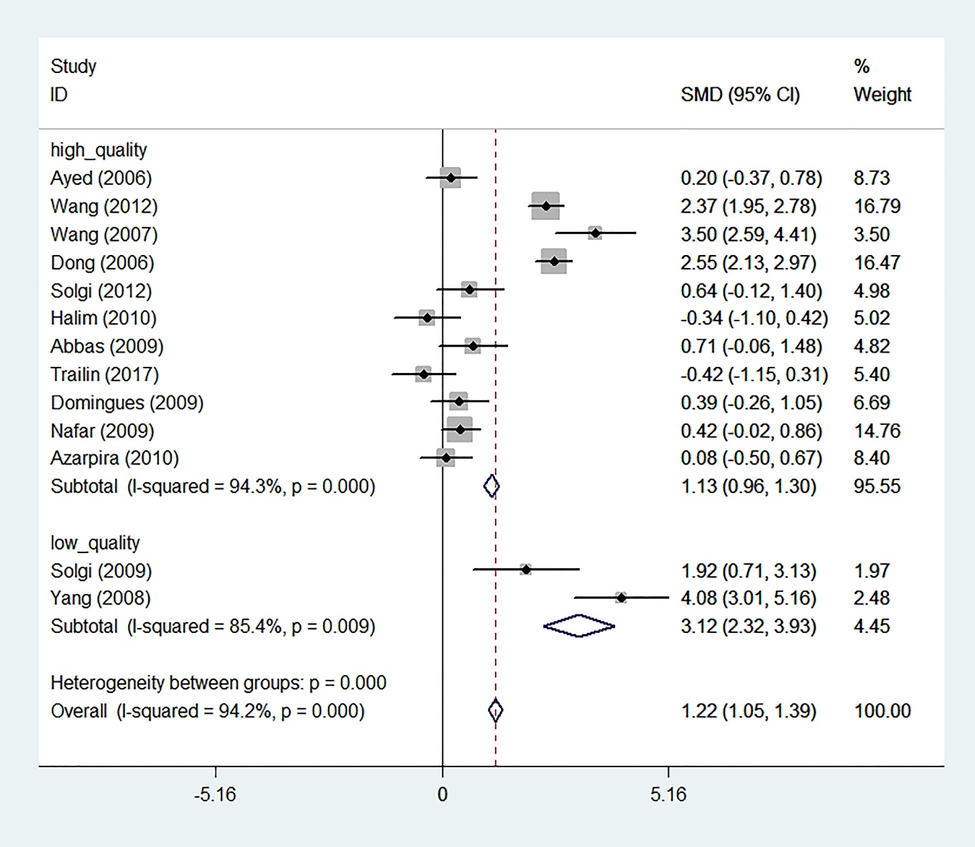


Supplementary figure 4. Subgroup by PRA in the 1-week post-transplant sCD30 group.


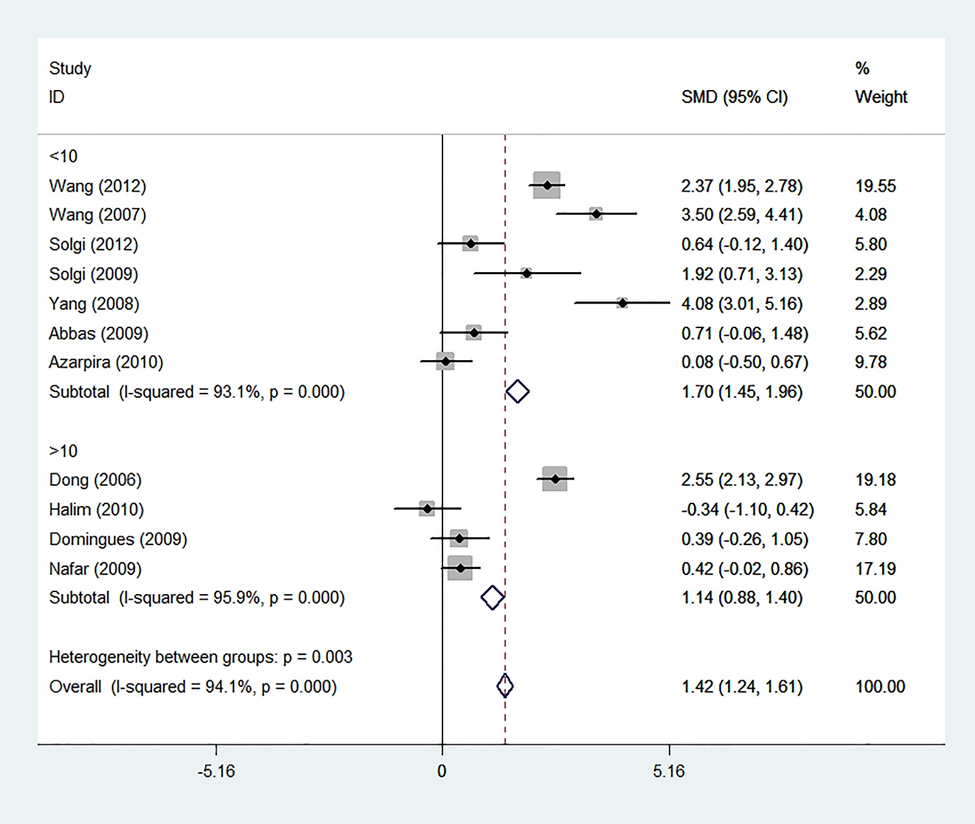


Supplementary figure 5. Meta-regression on age of rejection patients in the 1-week post-transplant sCD30 group.


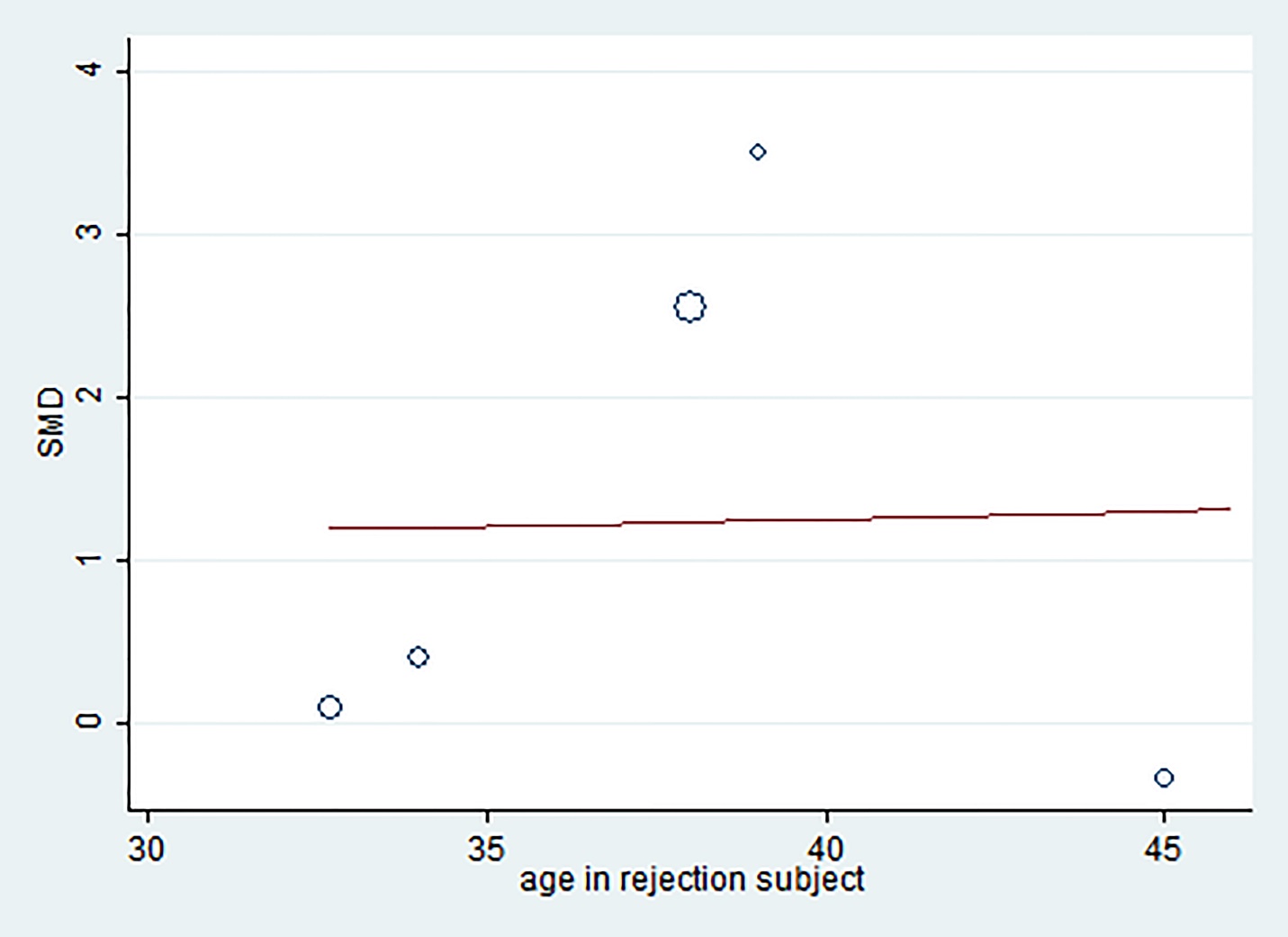


Supplementary figure 6. Subgroup by the number of transplanted kidneys in the 2-week post-transplant sCD30 group.


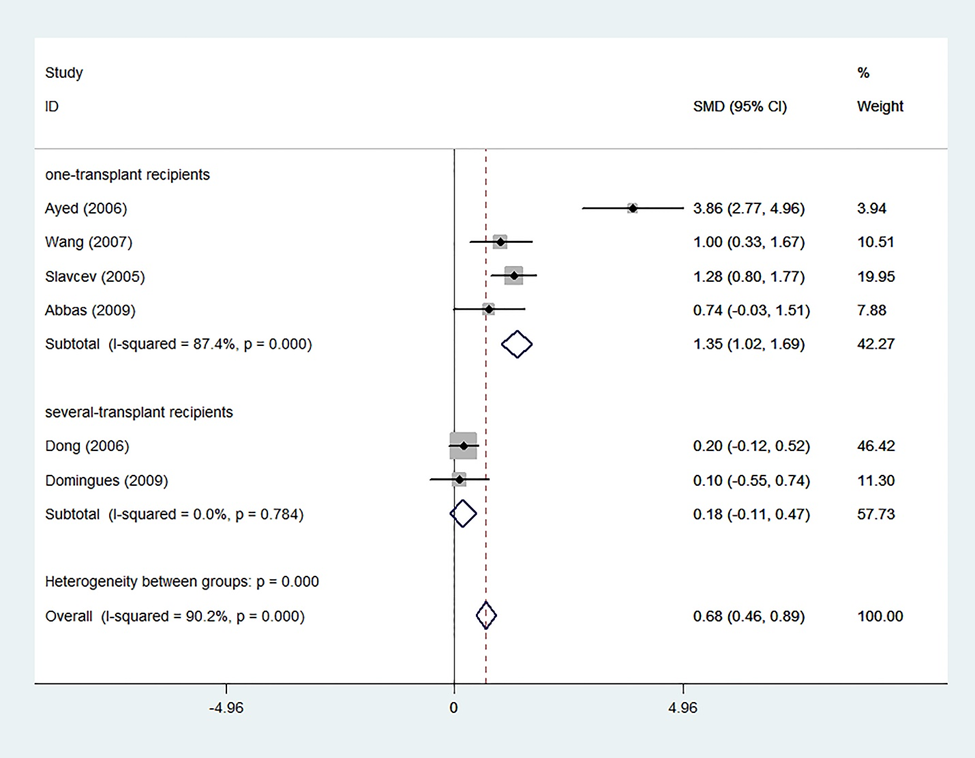


Supplementary figure 7. Subgroup by donor type in the 2-week post-transplant sCD30 group.


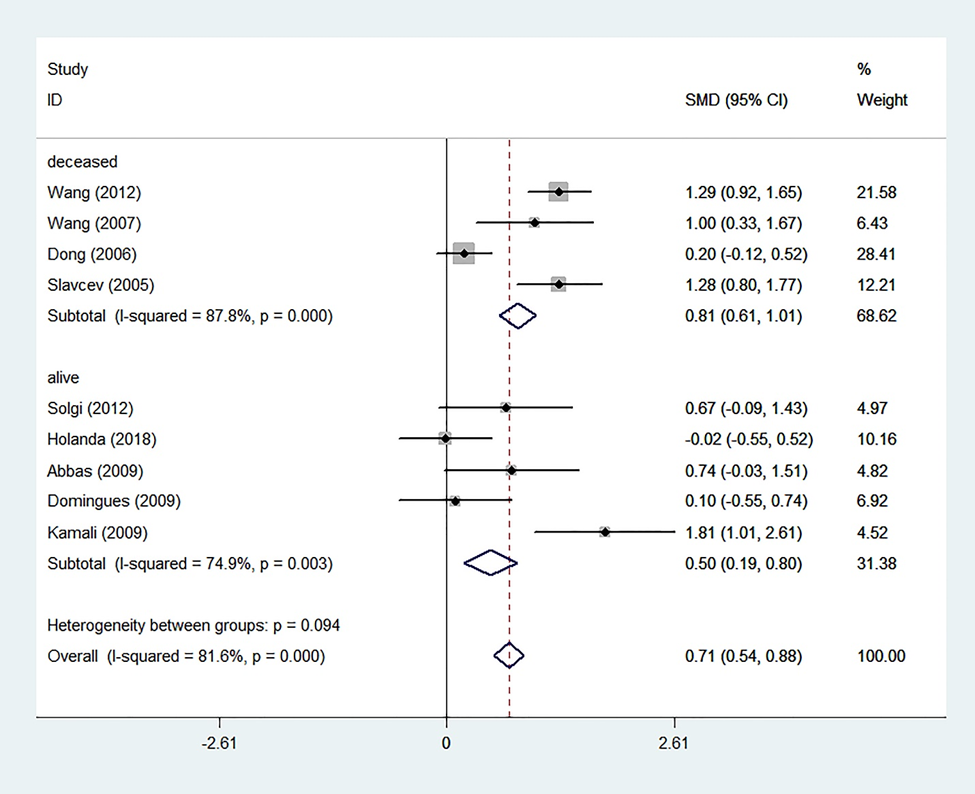


Supplementary figure 8. Subgroup by methodological quality in the 2-week post-transplant sCD30 group.


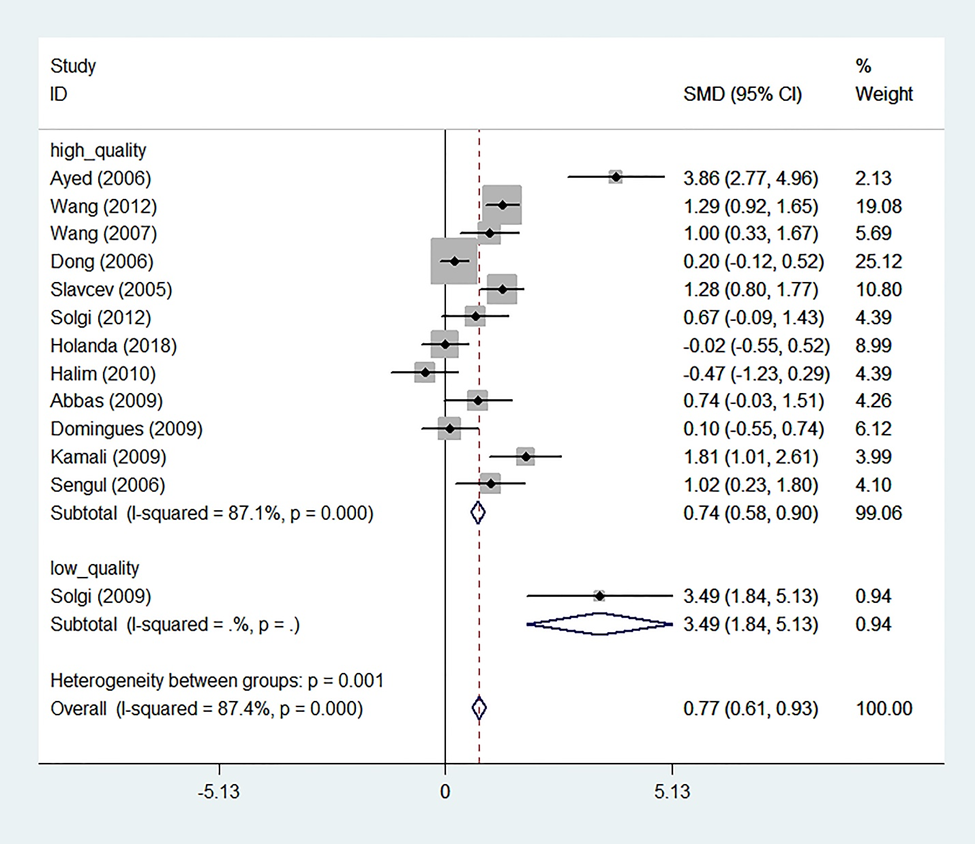


Supplementary figure 9. Subgroup by PRA in the 1-week post-transplant sCD30 group.


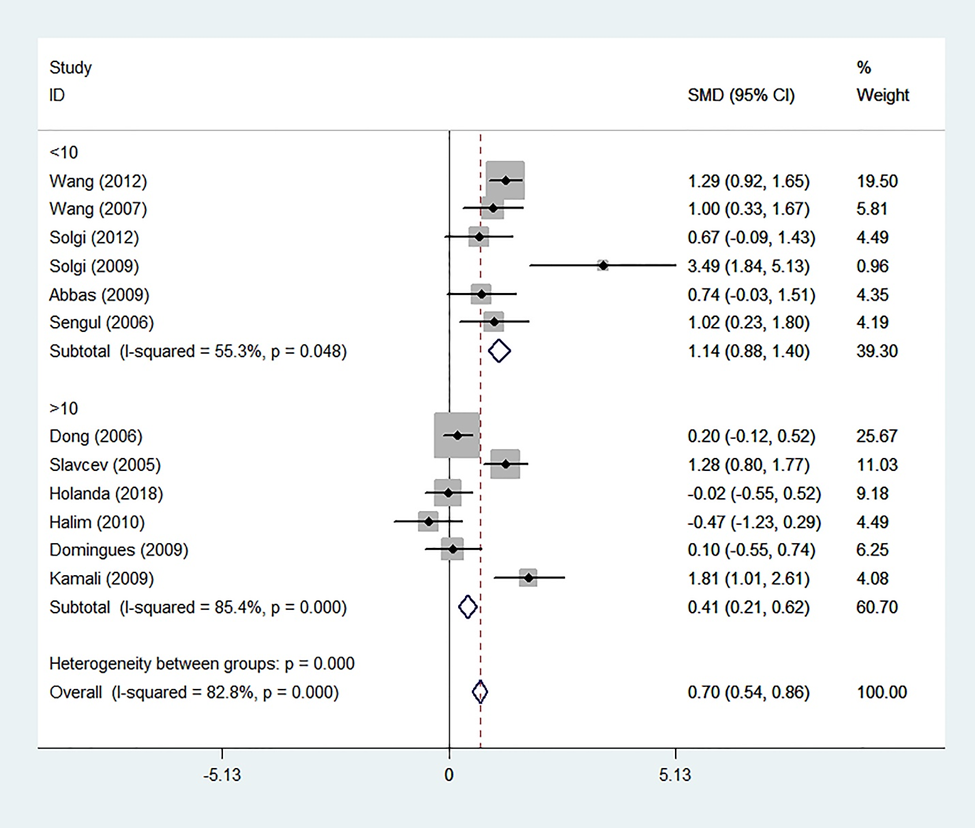


Supplementary figure 10. Meta-regression on age of rejection patients in the 1-week post-transplant sCD30 group.


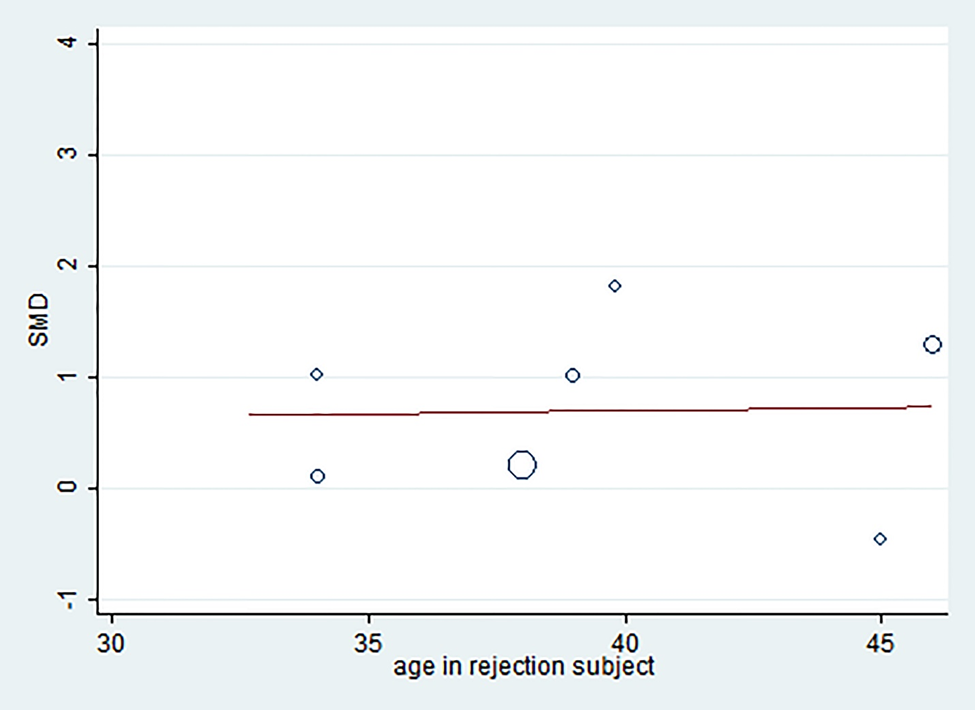


Supplementary figure 11. Meta-regression on CIT of rejection patients in the 1-week post-transplant sCD30 group.


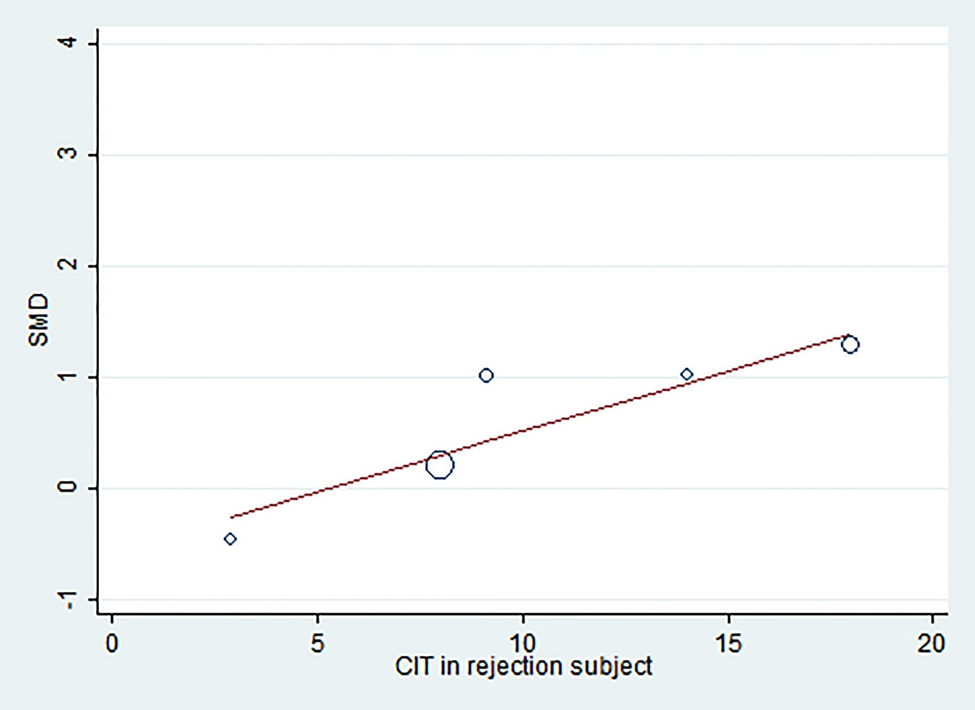


Supplementary figure 12. Association between sCD30 and acute rejection during three week after transplantation.


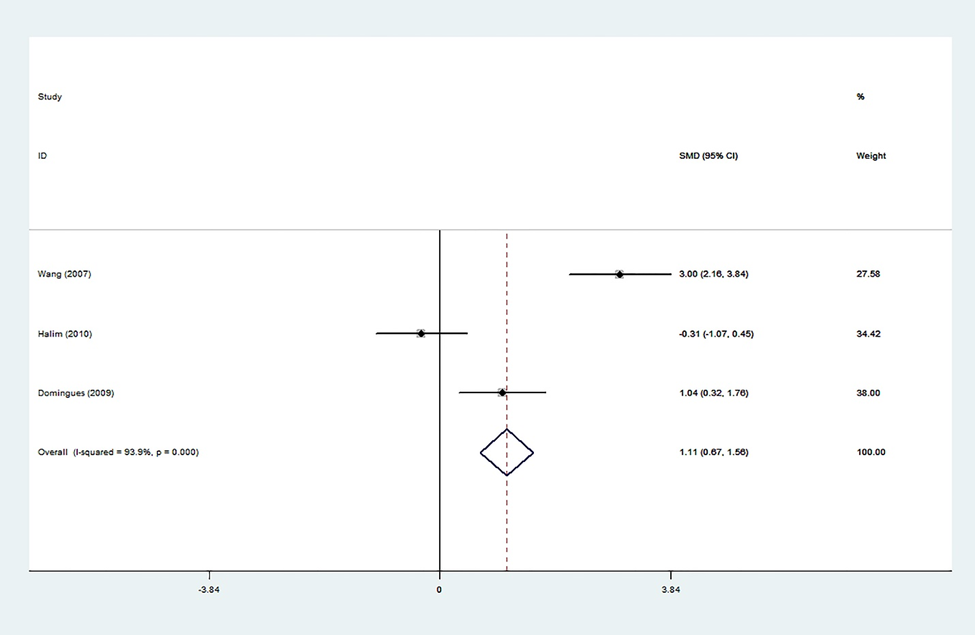


Supplementary figure 13. Association between sCD30 and acute rejection during four week after transplantation.


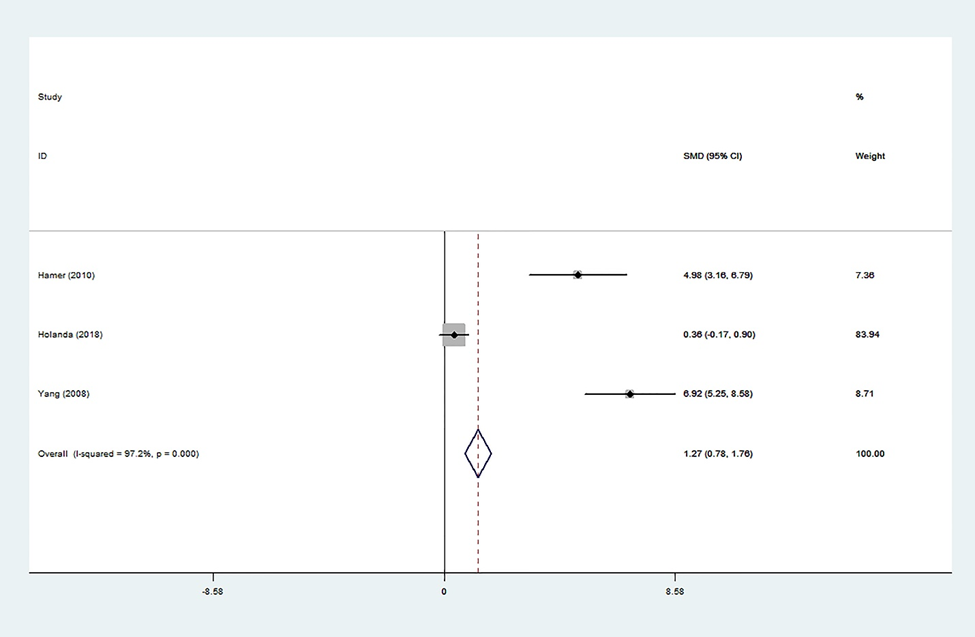


Supplementary figure 14. Association between sCD30 and acute rejection during one month after transplantation.


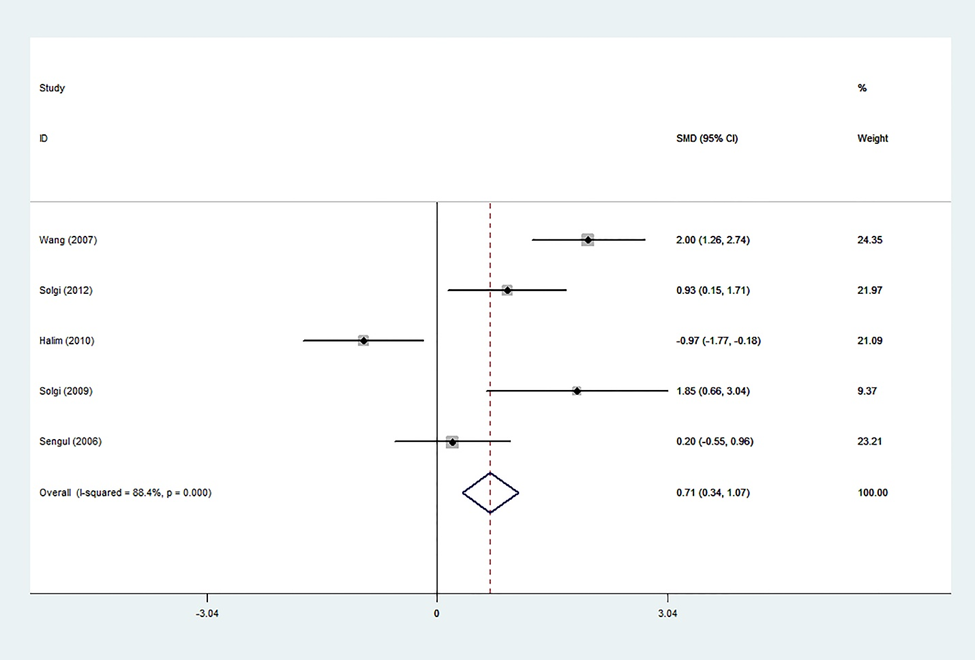

Supplement: Supplementary file 10 [file Data_Sheet_1.docx]
