# Supplementary material for: Soluble CD30, the Immune Response, and Acute Rejection in Human Kidney Transplantation: A Systematic Review and Meta-Analysis
Source: Front Immunol. 2020 Feb 28;11:295. doi: 10.3389/fimmu.2020.00295 (PMC7093023; doi:10.3389/fimmu.2020.00295)
Supplement: Supplementary file 11 [file Data_Sheet_2.docx]

**Newcastle-Ottawa Quality Assessment Form for Cohort Studies**

Note: A study can be given a maximum of one star for each numbered item within the Selection and Outcome categories. A maximum of two stars can be given for Comparability.

**Selection**

1) Representativeness of the exposed cohort

a) Truly representative ***(one star) (Random)***

b) Somewhat representative ***(one star) (non-random)(no restriction for choosing patients in specific groups )***

c) Selected group

d) No description of the derivation of the cohort

2) Selection of the non-exposed cohort

a) Drawn from the same community as the exposed cohort ***(one star)***

b) Drawn from a different source

c) No description of the derivation of the non-exposed cohort

3) Ascertainment of exposure

a) Validate measurement tool (e.g Eliza) ***(one star)***

b) Other measurement tool **(one star)**

c) No description

4) Demonstration that outcome of interest was not present at start of study

a) Yes ***(one star)***

b) No

**Comparability**

1) Comparability of cohorts on the basis of the design or analysis controlled for confounders

a) The study controls for age, sex and marital status ***(one star)***

b) Study controls for other factors (list) _________________________________ ***(one star)***

c) Cohorts are not comparable on the basis of the design or analysis controlled for confounders

**Outcome**

1) Assessment of outcome

a) Independent blind assessment (Two specialists) ***(one star)***

b) Biopsy as gold standard test ***(one star)***

c) Clinical signs

d) No description

e) Other

2) Was follow-up long enough for outcomes to occur

a) Yes (≥6 months) ***(one star)***

b) No (<6 months)

c) No description

Indicate the median duration of follow-up and a brief rationale for the assessment above:____________________

3) Adequacy of follow-up of cohorts

a) Complete follow up- all subject accounted for ***(one star)***

b) Subjects lost to follow up unlikely to introduce bias- number lost less than or equal to 20% or description of those lost suggested no different from those followed. ***(one star)***

c) Follow up rate less than 80% and no description of those lost

d) No statement

E-18

Thresholds for converting the Newcastle-Ottawa scales to AHRQ standards (good, fair, and poor):

**Good quality:** 3 or 4 stars in selection domain AND 1 or 2 stars in comparability domain AND 2 or 3 stars in outcome/exposure domain

**Fair quality:** 2 stars in selection domain AND 1 or 2 stars in comparability domain AND 2 or 3 stars in outcome/exposure domain

**Poor quality:** 0 or 1 star in selection domain OR 0 stars in comparability domain OR 0 or 1 stars in outcome/exposure domain
